# Supplementary material for: Online psychoeducation and assessment for borderline personality disorder as a first step of care: A pilot study assessing safety, feasibility, and mechanisms of change
Source: PLoS One. 2026 Aug 19;21(8):e0353836. doi: 10.1371/journal.pone.0353836 (PMC13489446; doi:10.1371/journal.pone.0353836)
Supplement: Supplementary file 1 [file pone.0353836.s004.pdf]

Study Progress

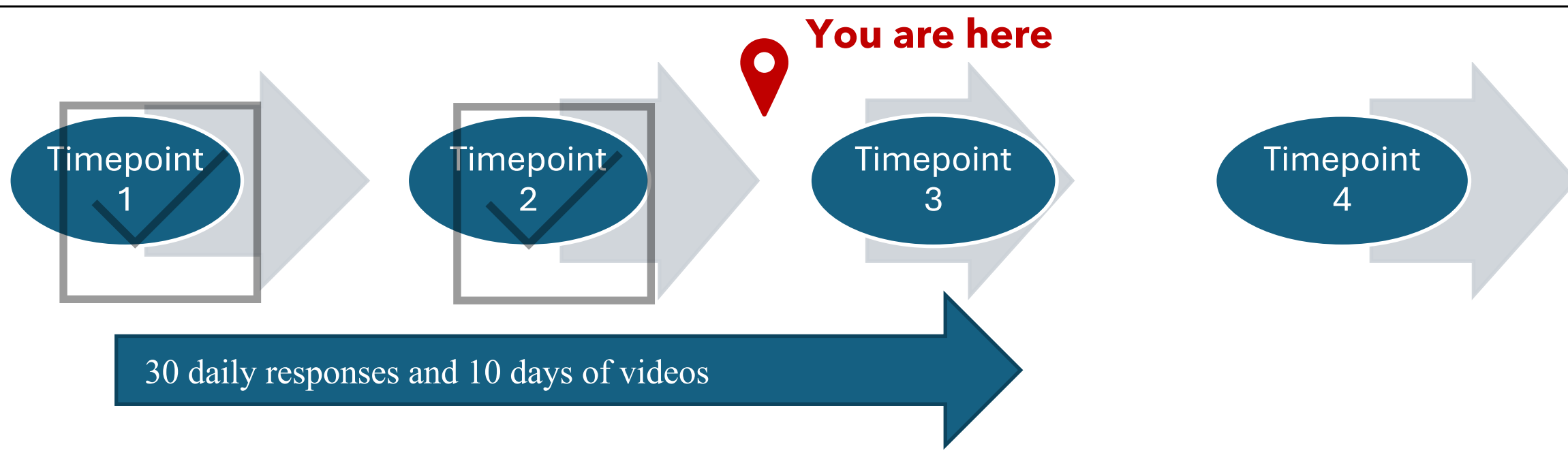

Your BPD Symptoms Feedback

In the charts below, you can see how you rated your BPD symptoms at Time 1 (Day 1 of this study) and at Time 2 (Day 15 of this study), and your overall symptom score.

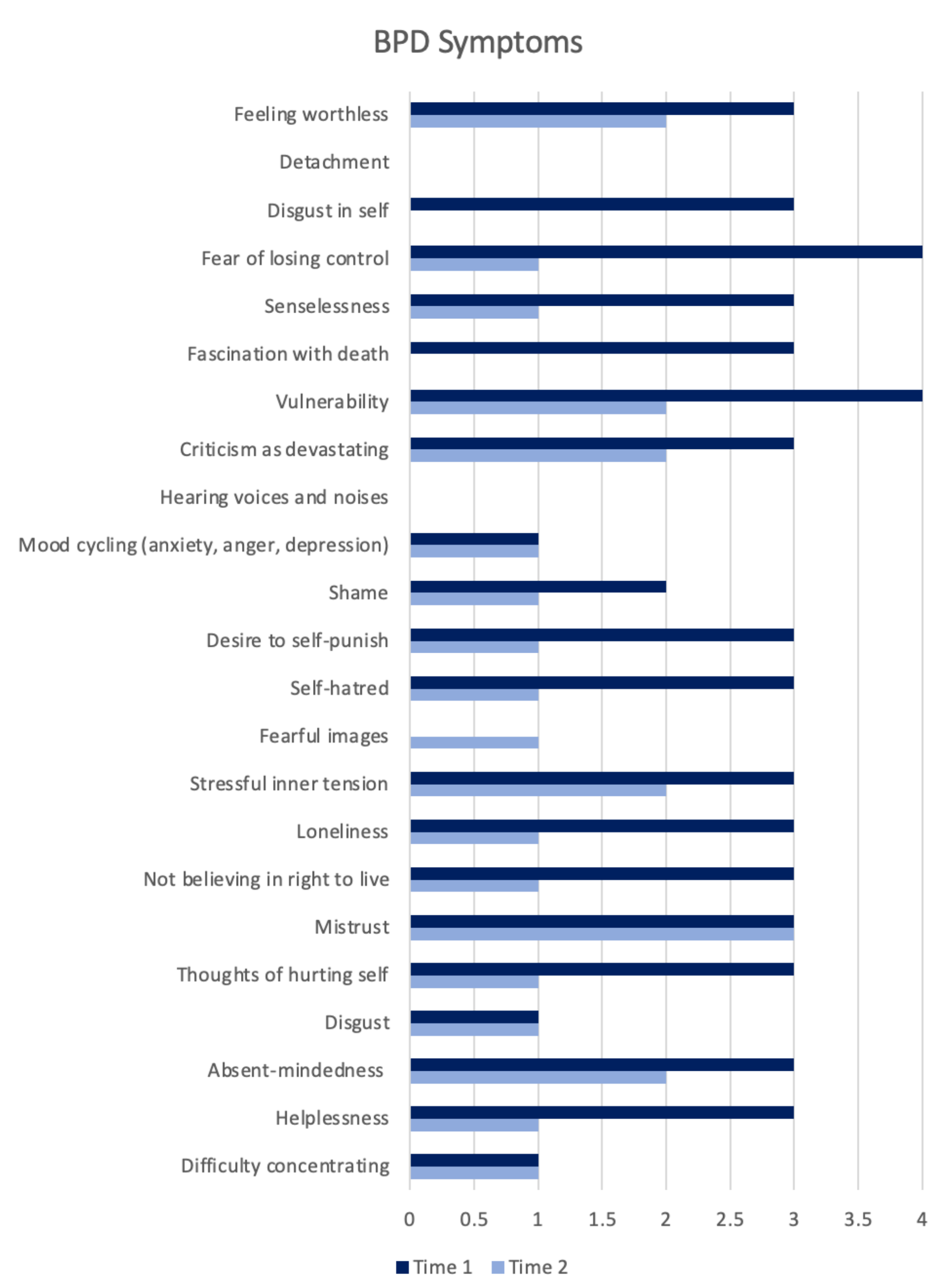

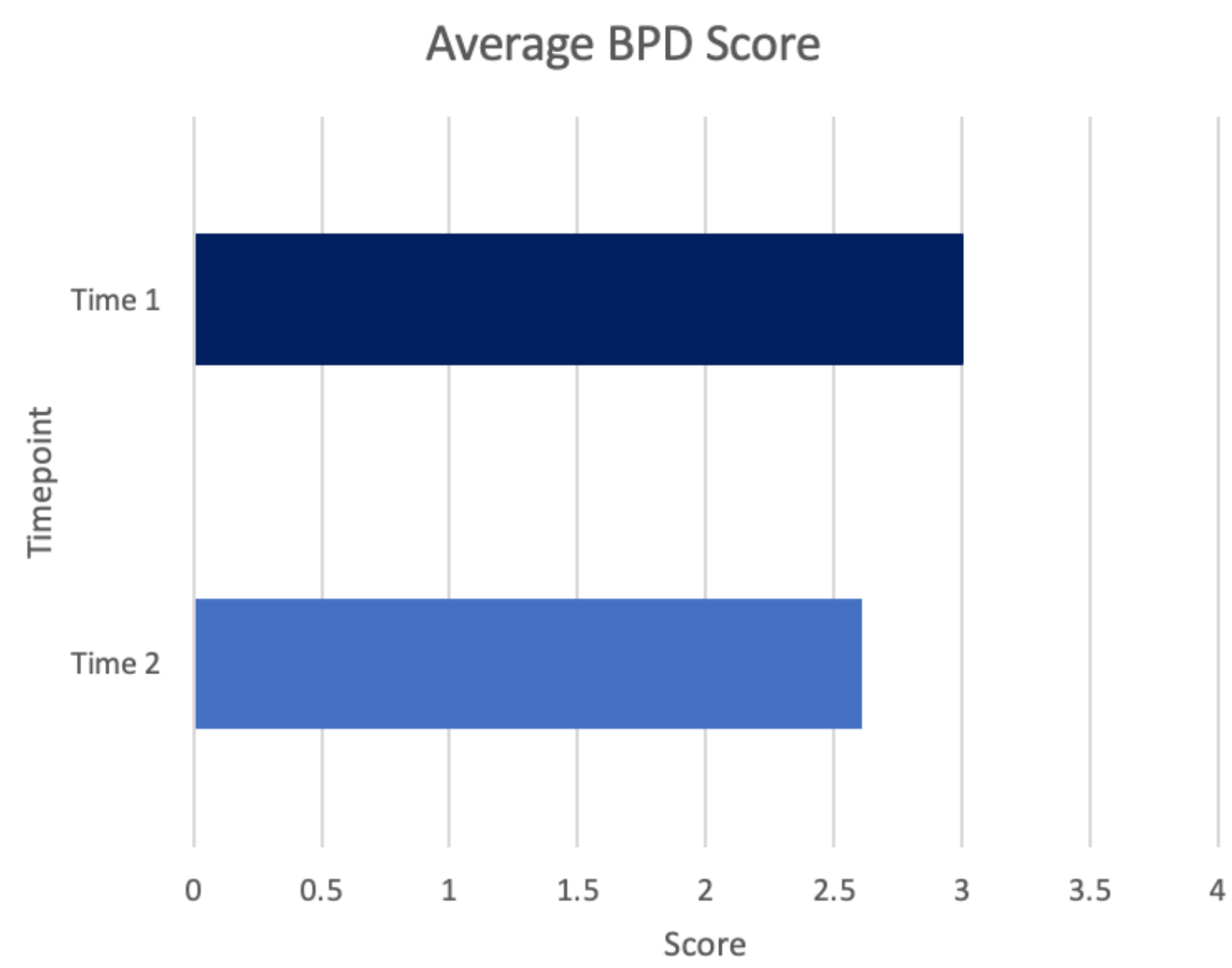

Your BPD Knowledge Feedback

In the chart below, you can see how you performed on a measure of your knowledge about BPD at Time 1 (Day 1 of this study) and at Time 2 (Day 15 of this study). A higher score indicates more knowledge about BPD. The maximum score is 13.

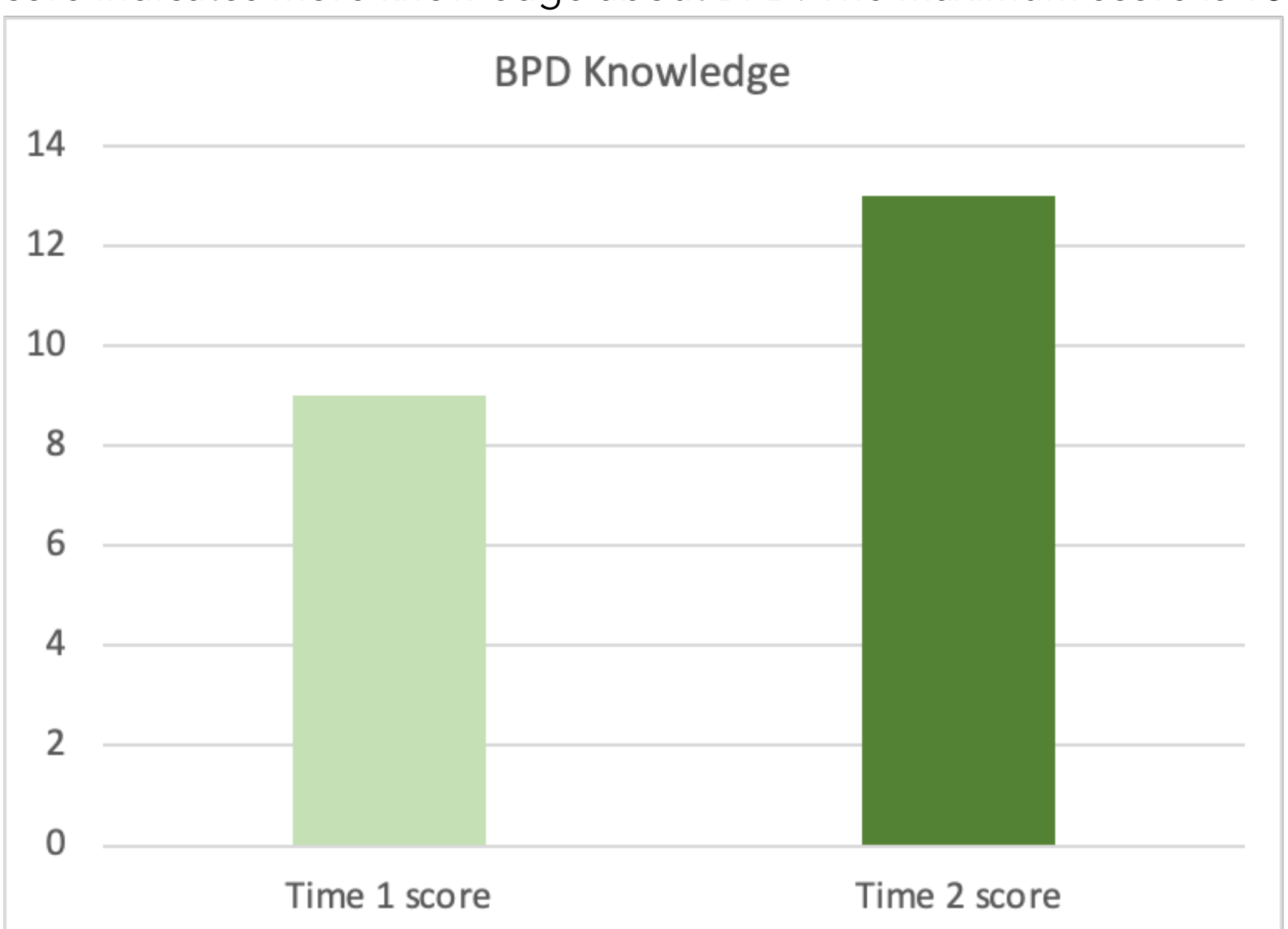

Your score on a BPD knowledge test improved at Time 2.

Your Depressive Symptoms Feedback

Below you can see the change in your overall depressive symptom score from Time 1 (Day 1 of this study) to at Time 2 (Day 15 of this study). A higher score indicates more depressive symptoms.

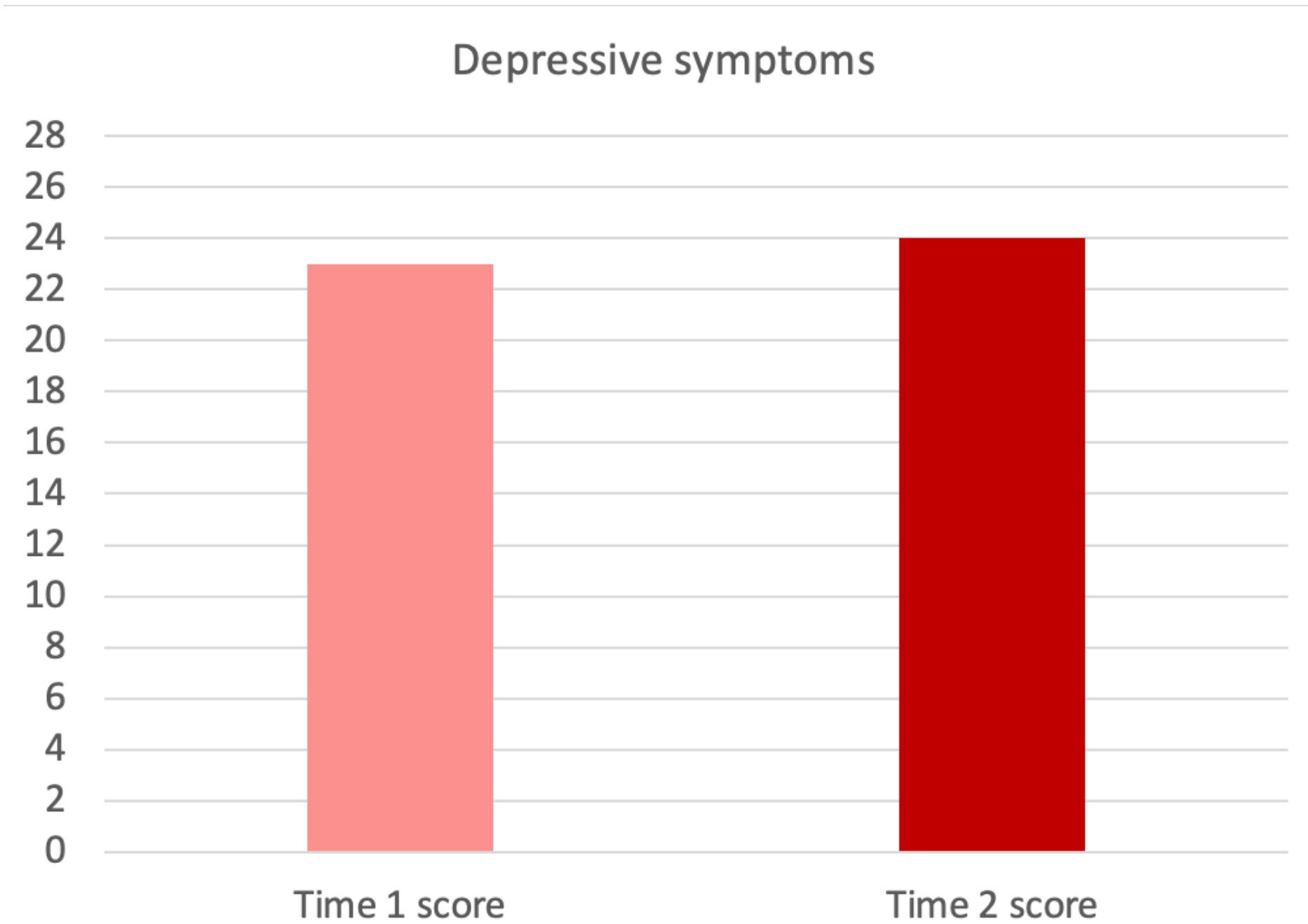

Your Loneliness Feedback

Below you can see how you rated your loneliness at Time 1 (Day 1 of this study) and at Time 2 (Day 15 of this study). A higher score indicates more loneliness symptoms.

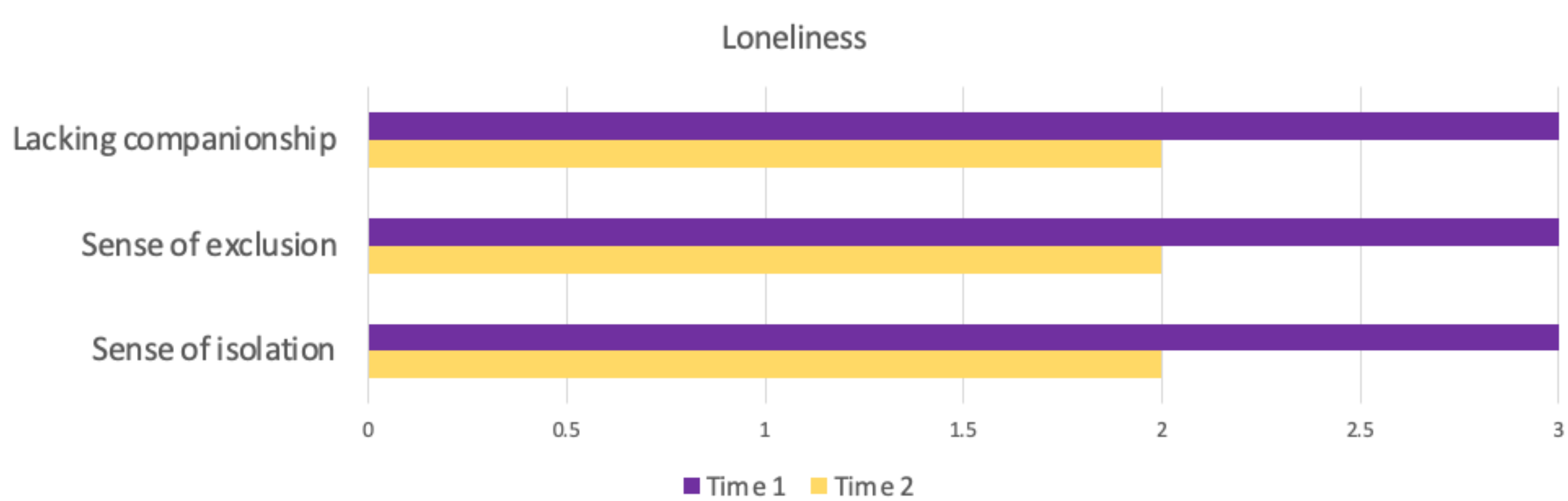

Your Personality Functioning Feedback

Below you can see how you rated your personality functioning at Time 1 (Day 1 of this

study) and at Time 2 (Day 15 of this study), as well as the change in your overall score. A *lower* score indicates *more stable* personality functioning.

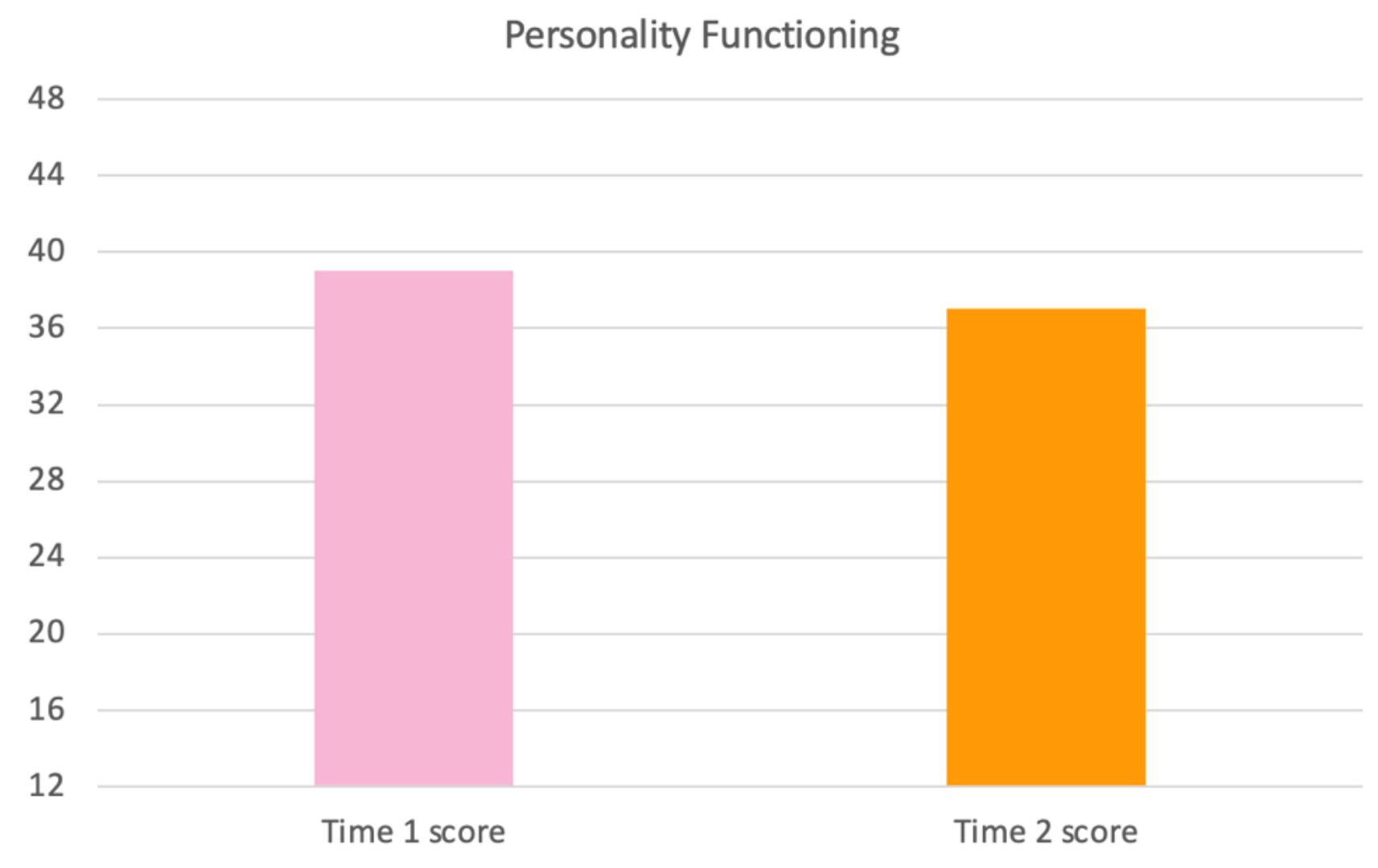

Your Interpersonal Feedback

Below you can see how many days you reported having had supportive and stressful interactions with others.

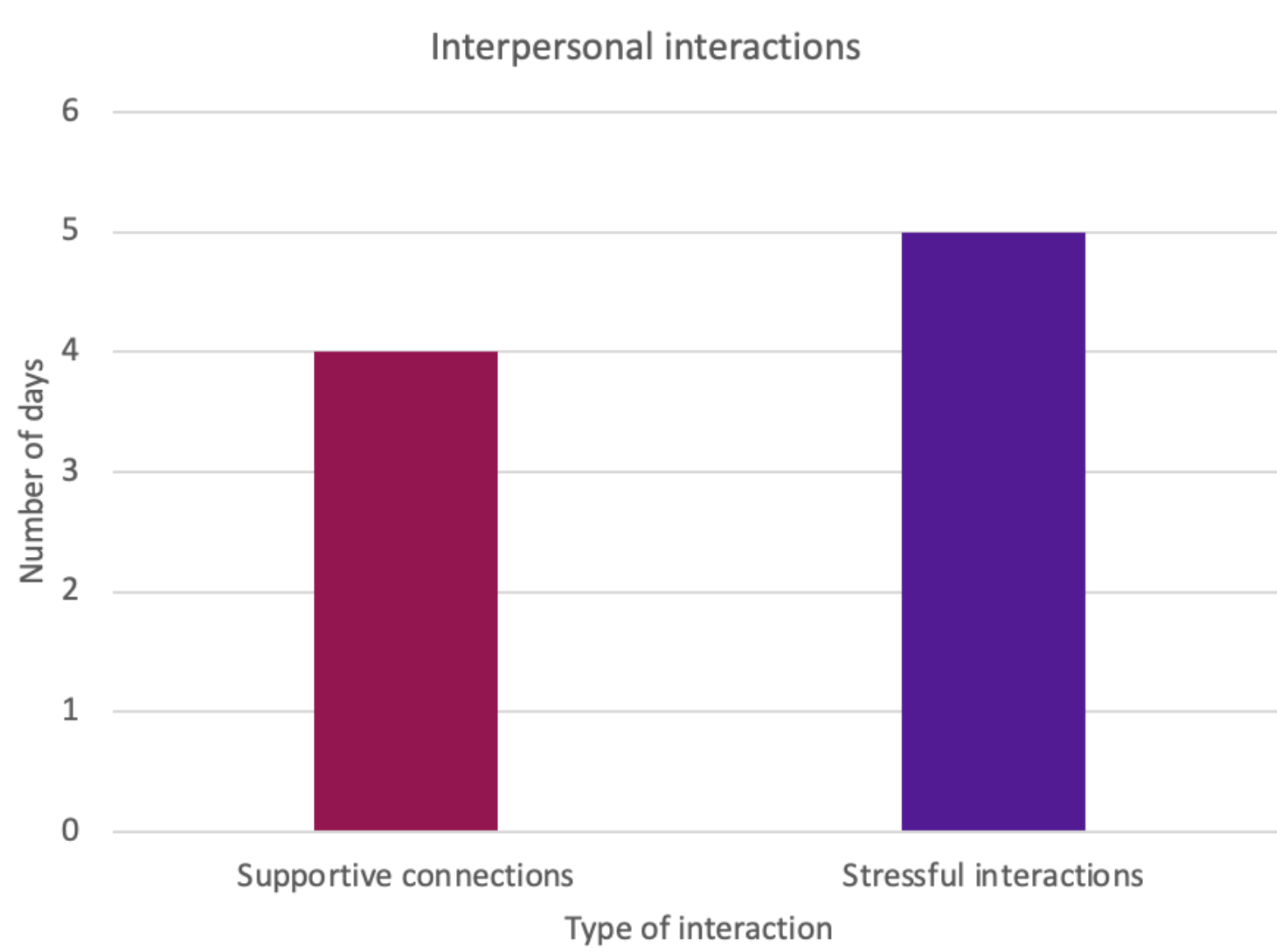

Your Cognitive Test Feedback

Please Note: *There are some limitations you should keep in mind as you look at your feedback. Each of these tests represents an area of active research - until we have gathered many results frompeople like you, we can't conclude anything about what participants' results mean. Furthermore, these kinds of results are only meaningful at the group level - that is, they may reflect patterns that apply on average and not necessarily to any one person. If you are surprised by your results on any of these tests, you shouldn't be concerned. There are many reasons why scores on this task might differ. For instance, differences in computer systems ordistractions while doing the task can affect results. These tasks were not designed to specifically assess you, and the scores can vary widely among the people who complete them.*

The Mountain/City Task

Sustained Attention and Response Inhibition

In this cognitive test, we assessed your sustained attention (i.e., your ability to concentrate) and your response inhibition (i.e., your ability to prevent yourself from performing an action).

Concentration

Your score is a measure of sustained attention. A higher score indicates better attention/concentration during the task.

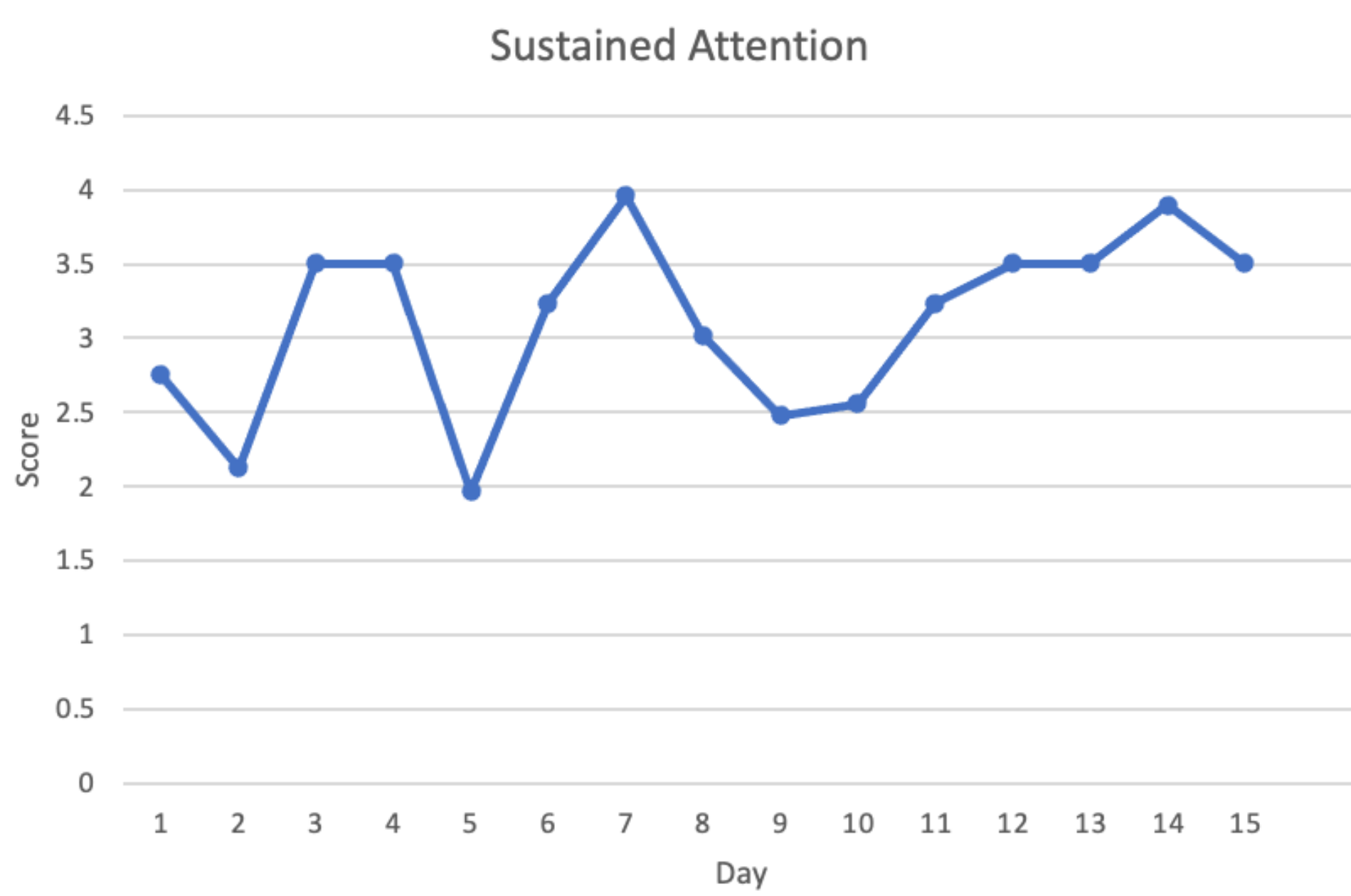

Response Inhibition

Your score indicates the number of times you pressed the button while a mountain scene was displayed (errors of commission). A higher percentage indicates poorer response inhibition.

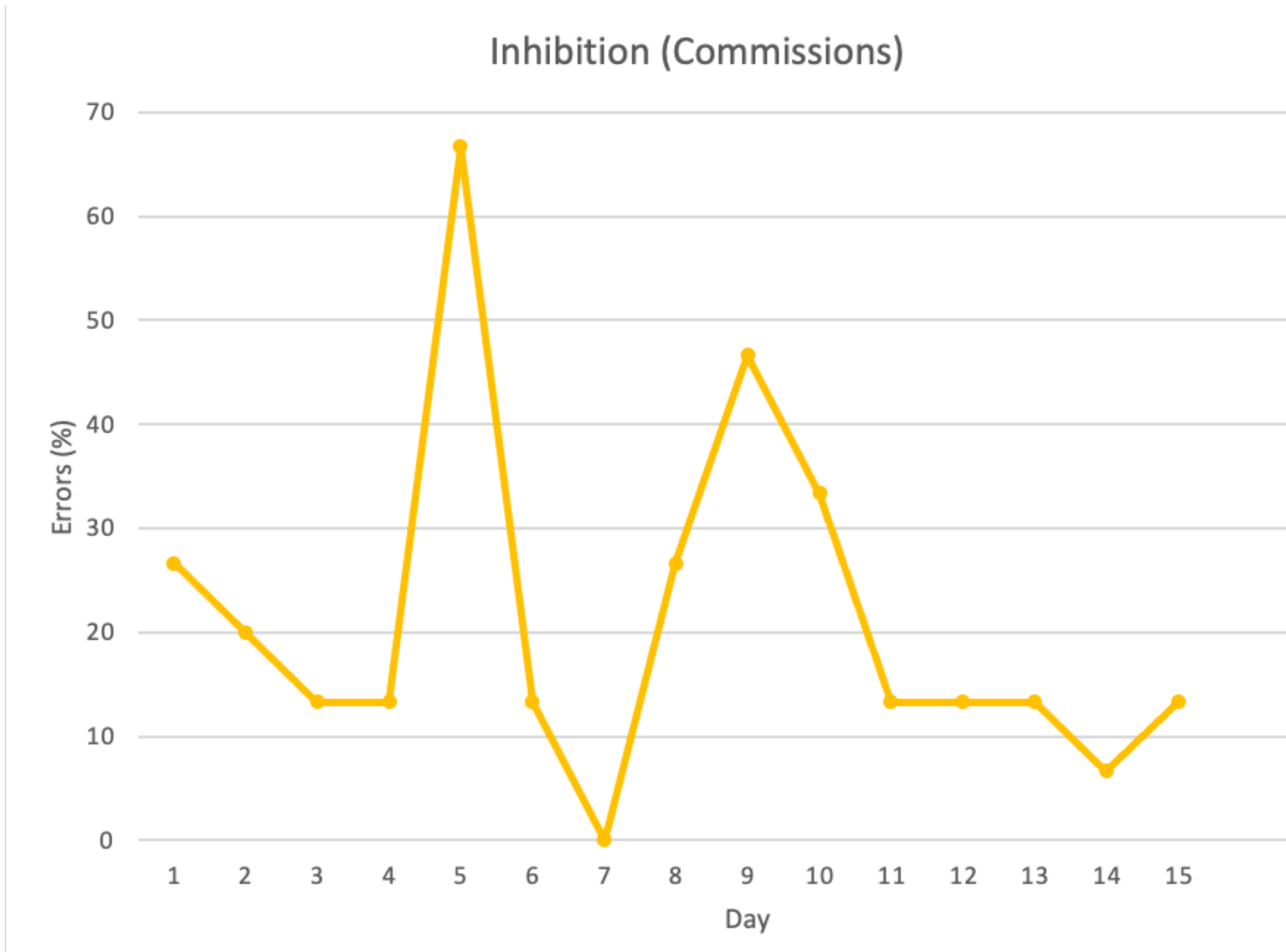

Here is how you scored relative to others:

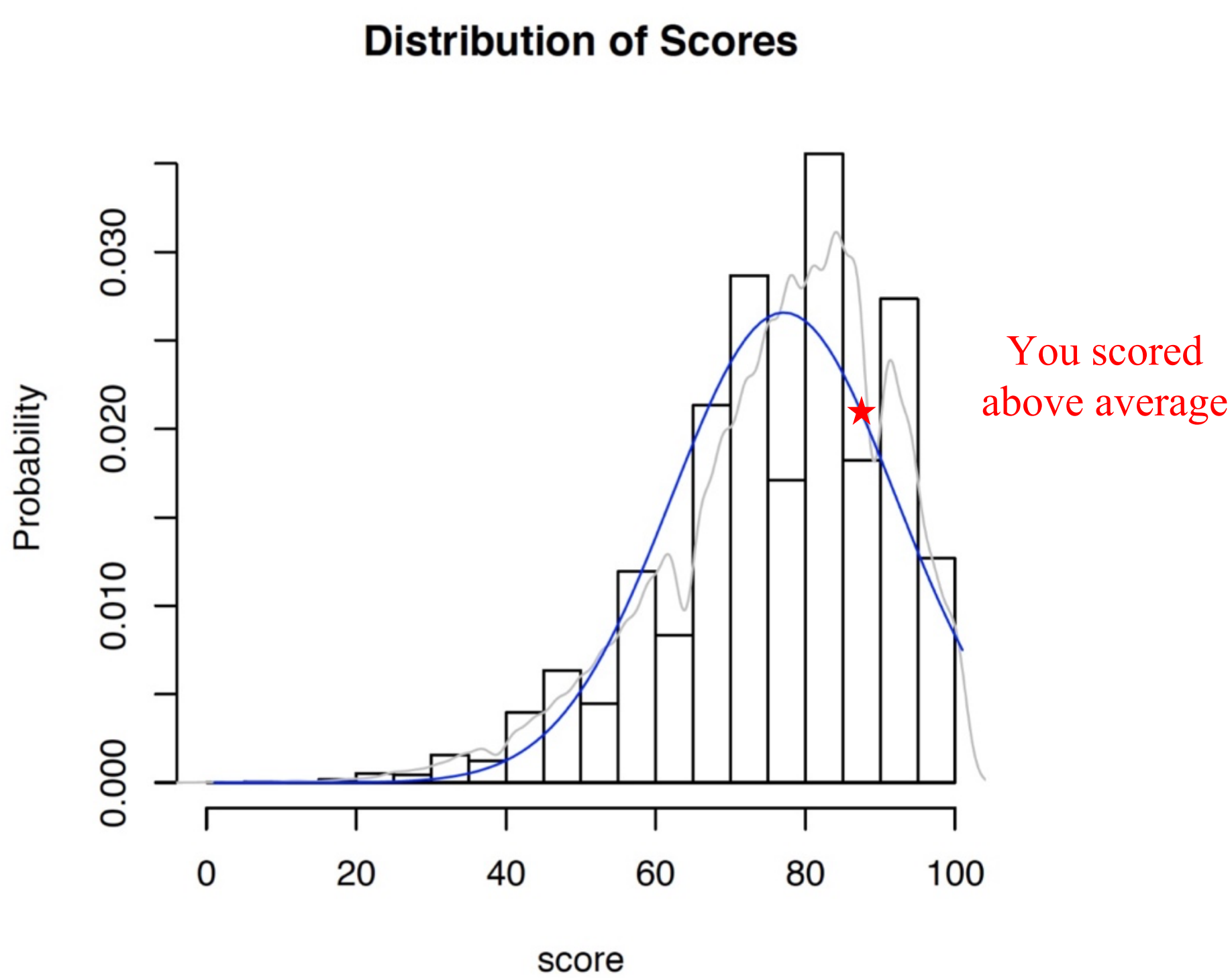

The Shape and Number Matching Task

Processing Speed and Visual Memory

In this cognitive test, we assessed your processing speed and your visual short term memory by asking you to match as many symbols and numbers as possible in 30 seconds.

Your score indicates the number of correctly matched digits and symbols. A higher score indicates better processing speed and visual memory.

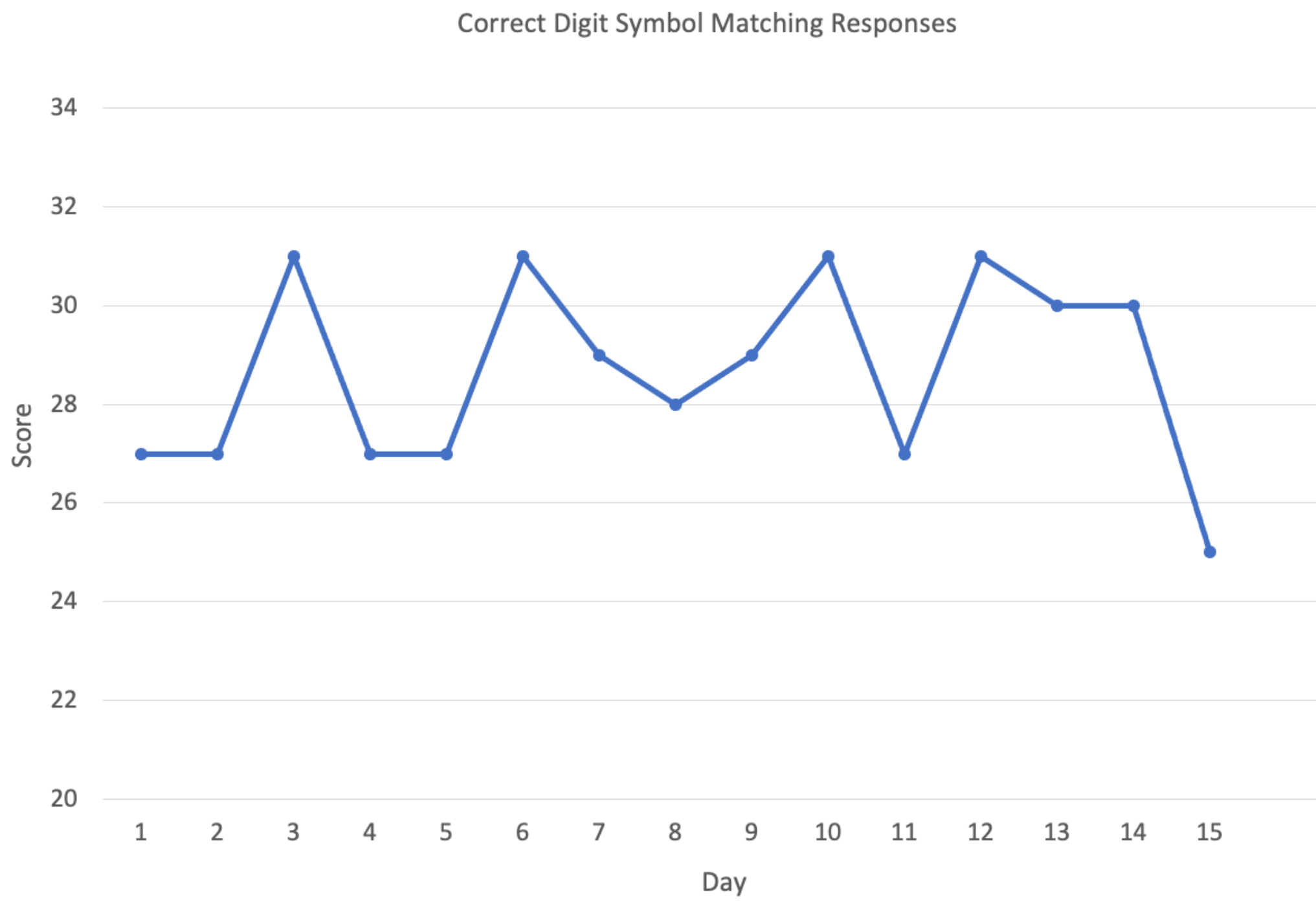

The Number Memorization Task

Memory

In this cognitive test, we assessed your memory by asking you to recall and type lists of digits in forward and or backward order.

<u>Forward</u>

Your score indicates the length of the longest list of digits you were able to accurately recall and type at least once. A higher score indicates better attention/concentration.

Time 1 (day 1 of this study)	Time 2 (day 15 of this study)
Score: **6** digits	Score: **6** digits

Your score remained the same between Time 1 and Time 2.

<u>Backward</u>
Your score indicates the length of the longest list of digits you were able to accurately recall and type at least once. A higher score indicates better working memory (a type of short-term memory for small amounts of information that are being used in the present moment to complete a cognitive task).

Time 1 (day 1 of this study)	Time 2 (day 15 of this study)
Score: **5** digits	Score: **5** digits

Your score remained the same between Time 1 and Time 2.

Summary

Between timepoints 1 and 2…

You showed significant or some improvement in:

- BPD symptoms such as shame, self-hatred, thoughts of hurting yourself, fear of losing control, experiencing criticism as devastating, fascination with death, desire to punish yourself, and feelings of worthlessness and helplessness.
- Overall BPD symptoms
- Sense of isolation
- Understanding your own and others' thoughts and feelings.
- Loneliness.

You remained stable in:

- Attention and concentration – you recalled the same number of digits.
- Short-term memory

Areas needing more support: The following symptoms appear to be higher at the second time point. Getting support in these areas could help.

- BPD symptoms such as fear of losing control, shame, loneliness, and difficulty concentrating
- Depression symptoms.
