# Supplementary material for: Online psychoeducation and assessment for borderline personality disorder as a first step of care: A pilot study assessing safety, feasibility, and mechanisms of change
Source: PLoS One. 2026 Aug 19;21(8):e0353836. doi: 10.1371/journal.pone.0353836 (PMC13489446; doi:10.1371/journal.pone.0353836)
Supplement: Supplementary file 2 [file pone.0353836.s006.pdf]

Patient Knowledge Test – Borderline Personality Disorder (PKT-BPD)

Developed by Lois Choi-Kain, Marcelo Brañas, Marcos Croci, and Julia Jurist

Correct answers are bolded

1. Which of the following is NOT a symptom of borderline personality disorder (BPD)?
    a. **Lack of remorse for actions**
    b. Desperate efforts to avoid abandonment
    c. Intense and reactive emotions
    d. Reckless self-endangering actions
2. How does interpersonal hypersensitivity explain why people with BPD have angry reactions towards others?
    a. People with BPD get angry because they don't value relationships
    b. People with BPD get angry because they can't tolerate frustration
    c. **People with BPD get angry when they feel threatened and feel they are losing support in an important relationship**
    d. People with BPD get angry because they are often mistreated by others
3. Understanding interpersonal hypersensitivity can help those with BPD in the following ways EXCEPT:
    a. It explains why you are self-destructive in relationships and towards yourself
    b. **It helps in understanding chronic depression that co-occurs with BPD**
    c. It helps you understand your reactions to others in times of distress
    d. It encourages you to work on connecting with others rather than expressing anger towards them or self-harming
4. How common is BPD?
    a. BPD is as common as major depressive disorder, affecting almost 20% of the public
    b. BPD is a rare condition in 1 out of 1000 people
    c. Approximately one in ten people will develop BPD in childhood
    d. **BPD occurs in 1-3% of people in the community, making it a fairly common mental health condition**
5. What causes BPD?
    a. Primarily trauma or other adverse life experiences
    b. Other mental illness such as substance use or mood disorder (e.g., bipolar)
    c. Primarily genetics (i.e., traits inherited from your biological parents)
    d. **Both traits at birth (biology) and adverse life experience**
6. What happens in the long term course of BPD?
    a. Most people with BPD who achieve remission from BPD will relapse, that is, they will re-experience symptoms of BPD after being in remission

    b. More people achieve recovery than remission. In other words, people are more likely to have a full-time job and stable relationships than to have symptoms of BPD
    c. Few people achieve symptomatic remission from BPD over 10 years, specifically, self-harm will remain common for most people
    d. **The majority of people achieve remission over 10 years but a smaller proportion achieve recovery (i.e. full-time work or school and at least one meaningful relationship outside of family)**
7. If someone who has BPD also has anxiety or depression, which should be treated as a primary focus?
    a. Anxiety, since medications will improve it and therefore make social interactions easier
    b. Depression, since it causes individuals with BPD to behave in an angrily manner to others and have no energy to solve their problems
    c. **BPD, since studies have shown that improvement of BPD leads to less time to remission from anxiety and depression**
    d. All of them at the same time, since there are medications that can target all three disorders simultaneously
8. Which of the following is a key principle for taking care of yourself when you struggle with BPD?
    a. Getting a life that provides direction, opportunities to build self-esteem, and connection to others
    b. Thinking before acting, since acting impulsively is rarely effective and can be detrimental in the long run
    c. Having experiences that challenge your fears and low self-worth. These experiences correct your opinion about your abilities or how others will respond to you
    d. **All of the above**
9. If someone with BPD feels suicidal, the best first step is:
    a. Communicate to those around you how much emotional pain you are feeling
    b. **Use the crisis plan and find a way to connect with others as a positive distraction during a difficult time**
    c. Isolate yourself until you no longer feel suicidal
    d. Ignore it or understand the wish to die isn't serious
10. Something that all treatments for BPD have in common is:
    a. They are all exclusively focused on relationships
    b. They involve prescribing medication targeting BPD symptoms
    c. They all involve using skills to be mindful
    d. **They offer an alternative, constructive way of thinking about your problems**
11. Which is true of BPD and medications?

  a. A higher number of medications leads to greater improvement
  b. Research has shown that most people with BPD are not on medications
  c. Since people with BPD have many and different types of symptoms, they should be on multiple psychiatric medications
  d. No medication has been consistently proven as a standalone treatment for BPD so none have been formally recommended by the Food and Drug Administration (FDA) for treating BPD

12. What type of treatment works best for BPD?
  a. Medications for depression and anxiety
  b. Hospitalization and emergency room visits when you need someone to talk to
  c. Psychotherapies that are structured and focused on problems related to BPD
  d. Intensive psychoanalytic psychotherapy (i.e., 4 times a week)
13. When you have BPD, it is essential to keep in mind all of the following EXCEPT:
  a. Since the intense emotional turmoil due to BPD can cloud your judgment, you should heavily rely on others to stay safe and get better
  b. While managing the symptoms of BPD is challenging, it is possible to get better and build a satisfying and meaningful life
  c. There are many treatments that work for BPD, and you should expect symptomatic improvement, otherwise the usefulness of treatment should be re-evaluated
  d. It is a common condition, so you are not alone. BPD is caused by both biology and stressful experiences so it is not your fault, though it is your responsibility to optimally manage your condition
