# Supplementary material for: Online psychoeducation and assessment for borderline personality disorder as a first step of care: A pilot study assessing safety, feasibility, and mechanisms of change
Source: PLoS One. 2026 Aug 19;21(8):e0353836. doi: 10.1371/journal.pone.0353836 (PMC13489446; doi:10.1371/journal.pone.0353836)
Supplement: Supplementary file 3 [file pone.0353836.s007.pdf]

Additional comments

1. BPD diagnosis
   a. How many participants were formally diagnosed vs self-diagnosed?
   b. Across recruitment sources, how was diagnosis confirmed (structured interview, clinician report, solely self-report)?

2. The CONSORT diagram excludes DBT/MBT, but the text states inclusion criteria were later expanded to allow some prior exposure. Please reconcile this discrepancy.
   a. What were the definitions of “partial” versus “full” exposure?
   b. How was adherence to previous therapy determined?

3. Considering BPD severity is a strength of this study.
   a. How were the “high” vs “low” categories defined?
   b. What was the pre-determined threshold?

4. How was it verified that participants watched the videos (clicks, time spent, self-report)?
   a. Could videos be watched multiple times?
   b. If a participant missed a day, could/did they watch two videos the next day?

5. Feedback is a convenient term for the additional component the feedback group received, but can it truly be considered purely informational – or did it likely function as an intervention alongside the videos? (particularly in relation to improvements in LPFS scores for the feedback group)

6. The BPD knowledge assessment appears closely tied to intervention content, raising potential “teaching to the test” concerns, especially since the 13-item multiple-choice measure was developed by the study team based on the BPD psychoeducational videos.
   a. Is there any reliability or psychometric data, or is this a non-validated measure?
   b. Were ceiling effects considered or observed?
   c. Was participant self-directed education measured? (i.e., research participants did unrelated to the study)

7. Defining safety solely as ED visits, hospitalization, or residential care may be limited for a BPD population.
   a. Were individual “high-risk” behaviors tracked separately (e.g., could self-harm be analyzed as its own variable, or was it combined with other high-risk behaviors)?
   b. Is including step-up to residential care appropriate, given that not all programs allow phone access, which could affect attrition?

8. Treating ordinal responses as integers and averaging across observations for the regression linking BSL change to time spent alone may be a crude approximation. Please justify this approach or consider alternatives that respect the ordinal nature, such as ordinal mixed models or cumulative link models.

9. Attrition is substantial (27 participants, 32.9%). Dropout may not be a robust proxy for a measure of acceptability, since participants may have discontinued for a variety of reasons.

10. P-value for gender reportedly comes from ANOVA, but gender is categorical → chi-square would be more appropriate.
