# Supplementary material for: Online psychoeducation and assessment for borderline personality disorder as a first step of care: A pilot study assessing safety, feasibility, and mechanisms of change
Source: PLoS One. 2026 Aug 19;21(8):e0353836. doi: 10.1371/journal.pone.0353836 (PMC13489446; doi:10.1371/journal.pone.0353836)
Supplement: Supplementary file 5 [file pone.0353836.s009.docx]

Dear Editors of PLOS One and Reviewers:

We thank you for your thorough and thoughtful review of our manuscript. We feel the feedback has improved and tightened our report. Please see the responses to each item below in **bold.**

Reviewer #1: This study addresses an important and challenging population and demonstrates several notable strengths. The inclusion of lived experience input is particularly valuable. Research with individuals with BPD is inherently difficult due to ethical considerations and risk management, which complicates recruitment, retention, and safety monitoring. Despite these challenges, the authors successfully implemented a fully online intervention and assessment protocol, demonstrating feasibility and acceptability in a population that is often hard to engage.

**Thank you for appreciating the challenges of a fully online randomized controlled trial. The fact that it was fully online with no face-to-face interaction or live virtual interactions after screening may explain some of the limitations in methodology brought up in the reviewer’s comments.**

Nonetheless, several methodological and reporting issues limit interpretation and generalizability. The authors appear aware of these limitations, and the preliminary data are promising. Please see the attached comments for specific revisions and clarifications regarding diagnosis verification, intervention adherence, measurement, safety monitoring, statistical approach, attrition, and potential confounds. I look forward to seeing this work progress.

Additional comments

1. BPD diagnosis a. How many participants were formally diagnosed vs self-diagnosed? b. Across recruitment sources, how was diagnosis confirmed (structured interview, clinician report, solely self-report)?

**All participants had to answer the following question during the Screening Questionnaire, “Were you diagnosed with borderline personality disorder (BPD) by a (mental) health care provider (e.g., primary care physician, psychologist, psychiatrist, social worker, or licensed mental health counselor) within the past six months?” All participants except for two self-reported being formally diagnosed by a health provider. Of these 80 participants, 48.8% were diagnosed by a clinician during inpatient psychiatric treatment, 46.2% were diagnosed by an outpatient mental health professional, 2.5% by a doctor in the emergency room, and 2.5% by a primary care physician. We had no way of verifying this information as we only had one live screening interview and no subsequent live interaction.**

**A Welch two-sample t-test showed no evidence of a significant difference in baseline BSL-23 scores between self-diagnosed and formally participants (t = 0.02, df = 2.24, p = .99). Mean scores were nearly identical between groups (Self: 2.30; Formal: 2.31). That said, the accuracy for this t-test is limited given the extremely low sample size in the self-diagnosed subgroup. Of the two participants who reported a self-diagnosis of BPD, one had a baseline BSL score of 2.43 and the other a score of 2.17, with both values falling within the high severity category.**

**Diagnosis was based solely on self-report, which we discuss as a limitation in the Discussion section of the manuscript on lines 784-790. However, baseline BSL-23 scores indicated that all participants scored above the typical range observed in healthy control samples, suggesting clinically relevant symptom levels. In this sample, the minimum baseline BSL-23 score was 0.74 compared to the 0.64 diagnostic threshold. We report the mean baseline BSL-23 score in the Limitations subsection of the Discussion section, and the minimum and maximum baseline scores are depicted in Table 1.**

**While this is not as reliable as a semi-structured interview method of diagnosis or clinical diagnosis, it is what we could complete with a total budget from this pilot study grant of $75K. Future definitive studies will employ more rigorous verification of diagnosis.**

2. The CONSORT diagram excludes DBT/MBT, but the text states inclusion criteria were later expanded to allow some prior exposure. Please reconcile this discrepancy.

**This discrepancy was noted in the legend of Fig 1 (CONSORT diagram). We also revised the legend for Fig 1 to clarify that the recruitment criteria were later expanded to extend eligibility beyond Massachusetts, given the online nature of the study.**

a. What were the definitions of “partial” versus “full” exposure? b. How was adherence to previous therapy determined?

**Research assistants performed all eligibility screening which was reviewed with the PI and first author to determine if exposure was significant or not. There were no means available to qualify or assess prior exposures since this trial did not conduct or supervise those treatments which were not video-taped or rated.**

**16 participants reported experience with evidence-based treatment (DBT, MBT, or TFP) in the Screening Questionnaire, but a total of 36 participants reported prior exposure to DBT (N=32), MBT (N=3), or TFP (N=1) during the formal Treatment History Inventory during the baseline assessment.**

**Provided below are what participants reported:**

- **DBT - 2 partial hospitalization programs outpatient 2 weeks (Salem hospital)**
- **DBT in outpatient programs**
- **DBT skills with outpatient therapist, never discussed BPD**
- **DBT in a partial program as a teenager**
- **DBT group (1 year) through an MGB hospital, not standard full package DBT**
- **did “some” DBT at McLean which does not offer adherent DBT in the general adult outpatient clinic, only groups**
- **4 participants did not provide information about what constituted their experience with prior EBTs in the Screening Questionnaire.**

**Partial exposure to evidence-based treatment was defined as limited engagement with a therapy without completing the full course as studied in prior RCTs. For example, one participant attended a two-week outpatient program that incorporated DBT-informed interventions, while another received DBT skills training from an outpatient therapist.**

**Information regarding how we defined full exposure to evidence-based treatment is reported in the Participants subsection of the Materials and Methods section of the manuscript (lines 212-214).**

3. Considering BPD severity is a strength of this study. a. How were the “high” vs “low” categories defined? b. What was the pre-determined threshold?

**Severity was based on baseline BSL scores, with a cut-off of 2.67. This information is reported in the Design subsection of the Materials and Methods section of the manuscript and was determined by the severity ranges indicated in prior studies establishing those standards (lines 238-239). Severity cutoffs for average score variable are: none/low (0-.28), mild (.28-1.07), moderate (1.07-1.87), high (1.87-2.67), very high (2.67-3.47), extremely high (3.47-4.0). Cutoff for diagnostic BPD is 1.50 compared to clinical controls and .64 compared to healthy controls.**

**These severity cutoffs were introduced and validated in the following paper: Kleindienst N, Jungkunz M, Bohus M. A proposed severity classification of borderline symptoms using the borderline symptom list (BSL-23). Borderline personality disorder and emotion dysregulation. 2020 Jun 1;7(1):11.**

4. How was it verified that participants watched the videos (clicks, time spent, self-report)? a. Could videos be watched multiple times? b. If a participant missed a day, could/did they watch two videos the next day?

**With our limited budget and low-tech method, we could not verified what participants watched or not, which is addressed as a limitation in the Limitations section of the Discussion on lines 790-792. However, the patient knowledge test of BPD information used assessed some items directly covered in the videos that suggest they had been watched. See answer to psychometric characterization of our knowledge test which was specifically developed for this study.**

**Yes, it is possible participants watched the videos multiple times. Yes, it was possible for participants to watch videos later. There was no timeframe in which video access was turned off.**

5. Feedback is a convenient term for the additional component the feedback group received, but can it truly be considered purely informational – or did it likely function as an intervention alongside the videos? (particularly in relation to improvements in LPFS scores for the feedback group)

**Please see supporting information S1 File Example of Feedback for your review so that you and readers can see the simplicity as well as impact of this automated feedback. These feedback documents were derived directly from participant self-report and cognitive testing data, and explanation of the data, and a summary of improvements, things that remained steady, and things that worsened. We hypothesized that measurement and feedback itself had therapeutic effects, and this part of the design is described in the prior protocol paper we published in this journal in 2023.**

6. The BPD knowledge assessment appears closely tied to intervention content, raising potential “teaching to the test” concerns, especially since the 13-item multiple-choice measure was developed by the study team based on the BPD psychoeducational videos. a. Is there any reliability or psychometric data, or is this a non-validated measure? b. Were ceiling effects considered or observed?

**We have added psychometric elaboration of this new measure (Patient Knowledge Test- Borderline Personality Disorder: PKT-BPD) we are including in Supporting Information S2 File BPD Knowledge Test. We have also added a reliability analysis using Cronbach’s alpha indicated suboptimal internal consistency across all timepoints (Time Point A: α = 0.50; Time Point B: α = 0.61; Time Point D: α = 0.62). Although reliability increased slightly over time, values remained below conventional thresholds for acceptable internal consistency. Cronbach’s alpha assumes one-dimensionality and item homogeneity. Violations of these assumptions, such as those common in knowledge-based assessments with heterogeneous content, can result in lower alpha values without necessarily indicating poor measurement quality (Edelsbrunner, Simonsmeier, & Schneider, 2025). See lines 327-338.**

**We also analyzed floor and ceiling effects as suggested. Our analysis indicated no floor effects at any timepoint, suggesting that the measure was not too difficult for participants and that minimum scores were not reached. In contrast, ceiling effects were observed and increased over time, indicating that a growing proportion of participants achieved the maximum possible score at follow-up assessments.**

**When examined by condition, the control group showed a modest ceiling effect at baseline (10.7%) that was not observed at later timepoints, suggesting stable and relatively limited BPD knowledge. The psychoeducation group demonstrated minimal ceiling effects at baseline (4%), which increased at Time Point B (27.8%) before decreasing at Time Point D (14.3%), indicating an initial increase in knowledge followed by a reduction in scores over time. The psychoeducation + feedback group showed the strongest ceiling effects, increasing from 3.45% at baseline to 28.6% at Time Point B and 35% at Time Point D, suggesting substantial knowledge gains throughout the study. We have added this in lines 504-516. The PKT-BPD measure can be found in Supporting Information S2 and the Table for the ceiling and floor effects can be found at S2 Table.**

c. Was participant self-directed education measured? (i.e., research participants did unrelated to the study)

**Self-directed psychoeducation was not tracked as we had no way of monitoring this aside from self-report.**

7. Defining safety solely as ED visits, hospitalization, or residential care may be limited for a BPD population. a. Were individual “high-risk” behaviors tracked separately (e.g., could self-harm be analyzed as its own variable, or was it combined with other high-risk behaviors)?

**The BSL-23 Supplement for Assessing Behaviors inquiries about the frequency of various high-risk behaviors in the past week, including suicide attempts and non-suicidal self-injury (NSSI). A composite risk variable was created using the NSSI and suicide attempt items from the BSL–23 Supplement, with higher scores reflecting greater frequency of these behaviors. Descriptive statistics for the composite risk variable are presented below and the manuscript’s Table 4, which is reported in the BPD Symptoms subsection of the Measures section (lines 347-350).**

**A linear mixed effects model with random intercepts for participant revealed that neither time (F = 0.42; p = .64), condition (F = 0.32, p = .88), nor the time x condition interaction (F = 1.14, p = .34) significantly predicted risk. In addition, a Poisson mixed effects model demonstrated that baseline risk did not significantly predict dropout (β = -0.011, p = .55), which is reported in the Change in Overall BPD Symptoms subsection of the Results (lines 542-547).**

**Descriptive Statistics for the Composite Risk Variable Across Time and Condition**

|  | | **Total** | **Control** | **Psychoed** | **Psychoed + Feedback** |
| --- | --- | --- | --- | --- | --- |
|  | | ***n*** | ***Mean (SD)*** | ***Mean (SD)*** | ***Mean (SD)*** |
| **NSSI & Suicide Attempt** | **Baseline** | **82** | **0.93 (1.63)** | **0.60 (1.04)** | **0.41 (0.91)** |
|  | **Time B** | **57** | **0.72 (1.13)** | **0.39 (0.78)** | **0.52 (1.75)** |
|  | **Time C** | **51** | **0.60 (1.14)** | **0.42 (0.80)** | **0.37 (0.60)** |
|  | **Time D** | **55** | **0.67 (1.11)** | **0.36 (0.63)** | **0.50 (1.79)** |

**Note. The number of participants at time points B and C does not match Fig 1 as some participants missed one assessment but later returned to complete a subsequent assessment.**

b. Is including step-up to residential care appropriate, given that not all programs allow phone access, which could affect attrition?

**In the present study, neither stepping up to residential care nor being hospitalized affected participation. The one participant who reported initiating residential treatment in the past 15 days and dropped out, continued in the study after entering residential treatment at time point B with completed data for that time point, but withdrew prior to time point C. Of the eight participants who reported hospitalization in the past 15 days, only one dropped out later on, specifically this participant was hospitalized prior to time point B and dropped out before time point D, having completed data through time point C.**

**Originally, one participant reported the same hospitalization event at both time points B and C. In addition, another participant reported entering residential care at time point B, but the reported date preceded completion of the screening questionnaire. Both participants’ responses were therefore recoded, resulting in changes to Table 2.**

**Fisher’s exact test indicated no statistically significant association between escalating care, either hospitalization or entering residential treatment (odds ratio = 1.3, 95% CI [0.02, 14.9], p = 1.00) and drop out status, though the estimate was imprecise, as reflected by the wide confidence interval due to the small number of events.**

8. Treating ordinal responses as integers and averaging across observations for the regression linking BSL change to time spent alone may be a crude approximation. Please justify this approach or consider alternatives that respect the ordinal nature, such as ordinal mixed models or cumulative link models.

**We would like to first clarify that the ordinal variable (time spent alone, EMA-assessed on a 1 to 4 scale) enters our regression as a *predictor*, with BSL change at Time C as the continuous outcome. The reviewer's suggested alternatives, cumulative link models and ordinal mixed models, are designed for ordinal *outcomes* and thus are not directly applicable here, which is why those methods were not adopted.**

**Second, the EMA time-spent-alone item is best understood as a coarsened continuous variable rather than a qualitatively ordered categorical one. Response options correspond to binned hours: 1 = "less than 1 hour," 2 = "1–3 hours," 3 = "4–6 hours," 4 = "7 or more hours." The category midpoints (~0.5, 2, 5, ~10 hours) are monotonically ordered and approximately linear with respect to the integer codes, so the 1-4 coding serves as a reasonable linear approximation of the actual hours spent alone. Averaging the integer codes across 30 observations therefore provides a close approximation to each participant's actual mean hours of time spent alone, and preserves the ordering of participants on their typical level of time spent alone.**

9. Attrition is substantial (27 participants, 32.9%). Dropout may not be a robust proxy for a measure of acceptability, since participants may have discontinued for a variety of reasons.

**Thank you for this point, we agree. This attrition rate is relatively high but still falls within the bounds of what is to be expected from patients with BPD (up to 42%) and is especially understandable given the lengthy nature of the study. See Iliakis EA, Ilagan GS, Choi-Kain LW. Dropout rates from psychotherapy trials for borderline personality disorder: a meta-analysis. Personal Disord. 2021 May;12(3):193-206. doi: 10.1037/per0000453. This is reference 64.**

**This information, comparing our dropout rate with the rate reported in other studies with patients with BPD, is detailed in the Discussion section of the manuscript (lines 652-655).**

10. P-value for gender reportedly comes from ANOVA, but gender is categorical → chi-square would be more appropriate.

**Thank you, it is a chi-square as explained in the Results section on lines 461-463. The count and frequency of female participants in each condition are reported in Table 1, but we have removed the p-values from Table 1 as recommended by another reviewer.**

Reviewer #2: This RCT takes an interesting and possibly very useful concept of using virtual tools to ameliorate the impact of BPD prior to receiving long term treatment.

Overall it is well written and comprehensive in describing methods, and results.
Most of the concerns I had have been addressed in the limitation section.

**Thank you for this positive review of the manuscript. We appreciate your focused feedback.**

One point to clarify is when you included those that had taken some form or duration of DBT and MBT did you consider the possible confounding effect on the response to the psycho education (with or without feedback)

**A Wilcoxon ranked sum test indicated that engagement in DBT, MBT, or TFP did not affect BSL scores or BPD Knowledge scores (our two primary outcomes) at any time point. Wilcoxon rank sum tests indicated no impact of receiving DBT, MBT, or TFP on BPD knowledge scores at time point B for the control group (*W* = 35.5, *p* = .82), the psychoeducation group (*W* = 17.5, *p* = .14), or the psychoeducation + feedback group (*W* = 29.5, *p* = .08) or time point D for the control group (*W* = 49, *p* = .75), the psychoeducation group (*W* = 12, *p* = .28), or the psychoeducation + feedback group (*W* = 25.5, *p* = .06). Wilcoxon rank sum tests indicated no impact of receiving DBT, MBT, or TFP on BSL-23 scores in the control, psychoeducation, and psychoeducation + feedback groups, respectively, at time point B (*W* = 35.5, *p* = .82; *W* = 22, *p* = .32; *W* = 62.5, p = .57), C (*W* = 46.5, *p* = .85; *W* = 14.5, *p* = 0.93; *W* = 50, *p* = 0.65), or D (*W* = 61, *p* = .64; *W* = 10, *p* = .19; *W* = 66.5, *p* = .21).**

**The table below which is now in the Supporting Information S3 Table presents the means and standard deviations for the two primary outcome measures, the BSL-23 and the GPM Knowledge of BPD Test. Among the 82 participants, 36 reported prior exposure to DBT, MBT, or TFP. 32 participants reported past exposure to DBT, 3 to MBT, and 1 to TFP. Partial exposure to evidence-based treatment was defined as limited engagement with a therapy without completing an adherent, full course of it as intended in the treatment manual. For example, one participant attended a two-week outpatient program that incorporated DBT-informed interventions, while another received DBT skills training from an outpatient therapist. The PI was responsible for ensuring that no participants had received adherent evidence-based treatment.**

**S3 Table. Comparison of people exposed to some DBT, MBT, or TFP versus people not exposed to any DBT, MBT, or TFP in terms of primary outcomes.**

|  |  | | **Exposure to**  **evidence based treatment**  **n = 36** | | **No exposure to evidence based**  **treatment**  **n = 46** | | **Wilcoxon Rank**  **Sum Test** | |
| --- | --- | --- | --- | --- | --- | --- | --- | --- |
|  |  | | **Mean** | **SD** | **Mean** | **SD** | **Test Statistic** | **p-**  **value** |
| **BSL-23** | **Baseline** | **Control** | 2.02 | 0.91 | 2.41 | 0.85 | 122.50 | .26 |
|  |  | **Psychoeducation** | 2.37 | 0.75 | 2.11 | 0.76 | 56.50 | .45 |
|  |  | **Psychoeducation with Feedback** | 2.35 | 0.90 | 2.51 | 0.75 | 115.00 | .78 |
|  | **Day 15** | **Control** | 2.09 | 1.08 | 1.92 | 1.08 | 35.50 | .82 |
|  |  | **Psychoeducation** | 2.54 | 0.74 | 2.07 | 1.15 | 22.00 | .32 |
|  |  | **Psychoeducation with Feedback** | 1.60 | 0.73 | 1.91 | 1.01 | 62.50 | .57 |
|  | **Day 30** | **Control** | 1.86 | 0.91 | 1.86 | 1.02 | 46.50 | .85 |
|  |  | **Psychoeducation** | 1.96 | 0.53 | 1.95 | 1.24 | 14.50 | .93 |
|  |  | **Psychoeducation with Feedback** | 1.38 | 0.51 | 1.65 | 0.88 | 50.00 | .65 |
|  | **Day 60** | **Control** | 1.80 | 1.03 | 2.09 | 1.08 | 61.00 | .64 |
|  |  | **Psychoeducation** | 2.62 | 0.40 | 1.86 | 0.90 | 10.00 | .19 |
|  |  | **Psychoeducation with Feedback** | 1.49 | 0.82 | 1.95 | 0.91 | 66.50 | .21 |

Also consider summarizing the introduction, it’s a bit long.

**Thank you for this feedback as we also felt it was long, but we wanted to be comprehensive rather than cursory, and this feedback enabled us to trim for readability.**

Reviewer #3: This is a feasibility and safety of a randomized controlled trial protocol of online psychoeducation and personalized feedback as an immediate first step of care for BPD.

Some comments for the authors:

1) While at this stage it might be late - consider title incoporates that this is randomised feasibility study - for searchability.

**Thanks for your suggestion. Our current title has the word feasibility and pilot in it.**

2) They are some differences between pilot and feasibility studies and these are used interchangeably in the manuscript - can this is be clarified. For example, feasibility studies usually assess parameters such as recruitment, retention, acceptability of intervention (.i.e as you have noted in lines 317 - 326). Pilot studies are essentially a study, e.g mimicing the larger study - however in this manuscript there seems to a combination of both with some statistical formal testing which seems inappropriate.

**Thank you for this granular level of feedback. Please review our protocol paper published in this journal in 2023. We understand your distinction, but our grant was expressly for a pilot study both assessing feasibility AND mimicking a protocol for a larger study which is necessary with high risk populations to move forward to definitive grant application, which was the pragmatic point of doing this trial in a methodologic progression of studies. Waiting longer by segregating these two types of studies appears impractical considering how difficult it is to obtain funding in the USA for BPD trials.**

3) There seems to be no justification of the number of people (sample size) for the study - even though they are planned significance tests

**A priori power analysis was conducted and reported in the protocol paper (Choi-Kain et al., 2023). It was determined that with a significance level of .05 and power of .8, the necessary sample size for each condition to detect a medium effect (Cohen’s d = .4) was 99 participants. However, given the aims of this pilot study as it was not powered to detect the expected small to medium effect sizes, we conducted exploratory analyses to detect emerging, preliminary signals.**

**Copied from the protocol paper for your ease to review this without further reading:**

**We expect a small to medium effect on BPD symptom severity, in line with effect sizes in the meta-analytic review of BPD’s effective interventions [2]. With a significance level of .05 and power of .8, the necessary sample size per condition to detect a small effect (Cohen’s d = .2) using two-sample t-test is 394, and to detect a medium effect (Cohen’s d = .4), n = 99. As a pilot study, this design is not adequately powered to detect the expected small to medium effect-size; therefore, we will conduct exploratory analyses in preparation for a future, large-scale investigation. Efficacy in this pilot trial will be estimated with 95% confidence intervals.**

4) Can there more information around the randomisation process (lines 243 - 248 talks about having fixed parameters to ensure balance - but this would be determined in the computergenerated scehedule, i.e having three groups with stratification as well - can more information be added to explain the rationale for this - or more clarity please. how was allocation concealment achieved?

**Block randomization was used to allocate participants to arms as they were recruited. Severity levels were considered as indicated in the response to Reviewer 1 Item 3 on severity levels. We show the patients were matched in terms of demographics and baseline parameters in the original draft Table 1. If you have suggestions on future methods of randomization for our grant application, we’d be open to suggestions.**

5) Assuming this is RCT feasibility study - there should be no between group differences at baseline since any differences are due to chance.

**Yes, we agree. There are no differences between groups at baseline as indicated by Tables 1 and 3 and lines 458-465 of the manuscript. Please note that, following Reviewer 1’s recommendation given our small sample size, p-values have been removed from Table 1. We nevertheless retained the statement in the manuscript noting that chi-square tests and ANOVAs were conducted and indicated no statistically significant baseline differences.**

6) Was there a statistical analysis plan, prior to final analyses,

**Yes, it is reported in the protocol paper (Choi-Kain et al., 2023). The analysis of the EMA data, however, was not included in the pre-registered plan and should therefore be regarded as preliminary and exploratory.**

7) Any independent committee reviewing/monitoring safety of these participants.

**The PI held the responsibility for continuous monitoring of data and safety of participants in the study. Data and safety monitoring took place continuously throughout the study’s duration. In the unlikely event of adverse events or serious adverse events, these events were documented and reported as required by the Partners Human Research Committees (PHRC) polity. As outlined by the PHRC policy, the principal investigator should report to the PHRC any of the following unanticipated problems and adverse events that occur: 1) during the conduct of the study, 2) after study completion, or 3) after participant withdrawal or completion. In such an event, reports will be submitted within 5 working days/7 calendar days of the date the investigator first becomes aware of the problem. At the time of the continuing review, we also provided the Partners IRB with a summary of any unexpected and related adverse events as well as any other unanticipated problems that occurred since the last continuing review. All other unanticipated incidents, experiences, information, outcomes, or other problems that indicate that the research places subjects at an increased risk of physical, psychological, economic, legal, or social harm than was previously known or recognized were submitted through Insight/eIRB as an Other Event. The PI ensured that the study adhered to the IRB-approved protocol and frequently monitored the overall progress of the study. The study staff ensured the accuracy and completeness of study materials and report all inconsistencies to the PIs.**

**No adverse events were reported to study staff.**

8) How was missing data handled?

**As described in the protocol paper, missing data were handled using multiple imputation, with results combined across imputed datasets using Rubin's rules for final inference. Imputation was performed under the assumption that data were missing at random (MAR). This information is reported in the manuscript’s Statistical Methods section on lines 427-431.**

9) Table 1 - all baseline symptoms, can you also report other metrics, i.e median range - since sample sizes are small, removed p-values

**We thank the reviewer for the suggestion. We have revised the table accordingly.**

|  | | **Total** | **Control** | **Psychoed** | **Psychoed + Feedback** |
| --- | --- | --- | --- | --- | --- |
| **Number of participants** | | 82 | 28 | 25 | 29 |
| **Mean age, years (SD)** | | 26.8 (8.77) | 27.7 (11.7) | 26.4 (6.97) | 26.3 (6.87) |
| **Gender (female)** | | 67 (81.7%) | 25 (89.3%) | 19 (76.0%) | 23 (79.3%) |
| **Ethnicity** | **African/Black** | 5 (6.1%) | 3 (10.7%) | 1 (4.0%) | 1 (3.4%) |
|  | **American Indian or Alaska Native** | 1 (1.2%) | 0 (0%) | 1 (4.0%) | 0 (0%) |
|  | **Asian** | 8 (9.8%) | 4 (14.3%) | 3 (12.0%) | 1 (3.4%) |
|  | **White** | 53 (64.6%) | 17 (60.7%) | 16 (64.0%) | 20 (69.0%) |
|  | **Other** | 6 (7.3%) | 4 (14.3%) | 1 (4.0%) | 1 (3.4%) |
|  | **Multiple** | 9 (11.0%) | 0 (0%) | 3 (12.0%) | 6 (20.7%) |
| **Education** | **HS or lower** | 12 (14.6%) | 5 (17.9%) | 2 (8.0%) | 5 (17.2%) |
|  | **Some college** | 37 (45.1%) | 11 (39.3%) | 15 (60.0%) | 11 (37.9%) |
|  | **Bachelor’s degree** | 22 (26.8%) | 8 (28.6%) | 5 (20.0%) | 9 (31.0%) |
|  | **Graduate degree** | 11 (13.4%) | 4 (14.3%) | 3 (12.0%) | 4 (13.8%) |
| **Baseline Symptoms** | |  |  |  |  |
| **BSL-23** | **Mean (SD)** | 2.28 (0.847) | 2.23 (0.884) | 2.17 (0.855) | 2.43 (0.813) |
|  | **Median [Min, Max]** | 2.39 [0.739, 3.74] | 2.17 [0.739, 3.65] | 2.22 [0.913, 3.65] | 2.48 [0.826, 3.74] |
| **PHQ-9** | **Mean (SD)** | 16.1 (5.44) | 15.4 (6.21) | 16.0 (5.01) | 16.8 (5.10) |
|  | **Median [Min, Max]** | 16.0 [1.00, 26.0] | 14.5 [5.00, 26.0] | 18.0 [1.00, 26.0] | 17.0 [6.00, 25.0] |
| **LPFS-BF** | **Mean (SD)** | 35.4 (6.01) | 34.1 (7.37) | 36.6 (5.17) | 35.7 (5.11) |
|  | **Median [Min, Max]** | 35.5 [21.0, 48.0] | 35.0 [21.0, 47.0] | 36.0 [24.0, 48.0] | 35.0 [23.0, 47.0] |
| **BPD Knowledge** | **Mean (SD)** | 9.94 (1.98) | 9.89 (2.06) | 9.28 (2.25) | 10.6 (1.48) |
|  | **Median [Min, Max]** | 10.0 [4.00, 13.0] | 10.0 [4.00, 13.0] | 9.00 [6.00, 13.0] | 11.0 [7.00, 13.0] |
| **BSL-23 Supplement 1** | **Mean (SD)** | 0.443 (0.663) | 0.571 (0.836) | 0.387 (0.567) | 0.368 (0.544) |
|  | **Median [Min, Max]** | 0.167 [0, 4.00] | 0.333 [0, 4.00] | 0 [0, 2.00] | 0 [0, 2.33] |
| **BSL-23 Supplement 2** | **Mean (SD)** | 0.477 (0.443) | 0.469 (0.526) | 0.495 (0.345) | 0.470 (0.445) |
|  | **Median [Min, Max]** | 0.375 [0, 2.63] | 0.375 [0, 2.63] | 0.500 [0, 1.13] | 0.375 [0, 1.50] |
| *Note:* BSL-23 = Borderline Symptom List – Short Version, PHQ-9 = Patient Health Questionnaire-9, LPFS = Level of Personality Functioning Scale-Brief Form, BSL-23 Supplement 1 = Borderline Symptom List Supplement: Items for Assessing Behavior Self-Harming Behaviors, BSL-23 Supplement 2 = Borderline Symptom List Supplement: Items for Assessing Behavior High-Risk Behaviors. | | | | | |

10) Table 3 - Primary outcomes - firstly maybe have a foot note for meaning og Time B, C, D and really the primary outcomes of the feasibility studies should be the ones mentioned, i.e recruitment etc - can this be clarified.

**We have revised Table 3 to include outcomes of feasibilities.**

| **Measures** | | | **Control** | **Psychoed** | **Psychoed + Feedback** |
| --- | --- | --- | --- | --- | --- |
| **BSL-23** | **Baseline** | Mean (SD) | 2.23 (0.88) | 2.25 (0.75) | 2.43 (0.81) |
|  | **Time B** | Mean (SD) | 1.99 (1.05) | 2.20 (1.05) | 1.78 (0.90) |
|  |  | Δ vs Ctrl [95% CI] | - | 0.154 [-0.489, 0.797] | -0.295 [-0.850, 0.260] |
|  |  | Cohen's d [95% CI] | - | 0.159 [-0.534, 0.853] | -0.326 [-0.947, 0.295] |
|  | **Time C** | Mean (SD) | 1.86 (0.94) | 1.95 (1.08) | 1.54 (0.74) |
|  |  | Δ vs Ctrl [95% CI] | - | 0.069 [-0.664, 0.802] | -0.403 [-1.073, 0.267] |
|  |  | Cohen's d [95% CI] | - | 0.072 [-0.702, 0.847] | -0.445 [-1.227, 0.337] |
|  | **Time D** | Mean (SD) | 1.97 (1.04) | 2.08 (0.85) | 1.74 (0.88) |
|  |  | Δ vs Ctrl [95% CI] | - | 0.246 [-0.256, 0.748] | -0.267 [-0.735, 0.201] |
|  |  | Cohen's d [95% CI] | - | 0.296 [-0.324, 0.916] | -0.330 [-0.917, 0.257] |
| **GPM Knowledge of BPD** | **Baseline** | Mean (SD) | 9.89 (2.06) | 9.44 (2.22) | 10.55 (1.48) |
|  | **Time B** | Mean (SD) | 9.33 (2.03) | 10.67 (2.11) | 11.38 (1.36) |
|  |  | Δ vs Ctrl [95% CI] | - | 1.653 [0.241, 3.065]* | 1.161 [-0.143, 2.465] |
|  |  | Cohen's d [95% CI] | - | 0.751 [0.113, 1.389]* | 0.553 [-0.044, 1.150] |
|  | **Time D** | Mean (SD) | 9.43 (2.20) | 10.00 (1.92) | 11.65 (1.39) |
|  |  | Δ vs Ctrl [95% CI] | - | 0.810 [-0.788, 2.408] | 1.251 [-0.050, 2.552] |
|  |  | Cohen's d [95% CI] | - | 0.355 [-0.364, 1.074] | 0.542 [-0.067, 1.150] |
| **Risk** | **Baseline** | Mean (SD) | 0.93 (1.63) | 0.60 (1.04) | 0.41 (0.91) |
|  | **Time B** | Mean (SD) | 0.72 (1.13) | 0.39 (0.78) | 0.52 (1.75) |
|  |  | Δ vs Ctrl [95% CI] | - | 0.239 [-0.733, 1.211] | 0.709 [-0.165, 1.583] |
|  |  | Cohen's d [95% CI] | - | 0.167 [-0.516, 0.850] | 0.490 [-0.119, 1.099] |
|  | **Time C** | Mean (SD) | 0.60 (1.14) | 0.42 (0.79) | 0.37 (0.60) |
|  |  | Δ vs Ctrl [95% CI] | - | 0.449 [-0.506, 1.404] | 0.991 [0.099, 1.883]* |
|  |  | Cohen's d [95% CI] | - | 0.330 [-0.391, 1.050] | 0.735 [0.097, 1.372]* |
|  | **Time D** | Mean (SD) | 0.67 (1.11) | 0.36 (0.63) | 0.50 (1.79) |
|  |  | Δ vs Ctrl [95% CI] | - | 0.304 [-0.539, 1.147] | 0.726 [-0.021, 1.473] |
|  |  | Cohen's d [95% CI] | - | 0.214 [-0.414, 0.843] | 0.501 [-0.050, 1.052] |
| *Note:* Outcome measures are depicted above. Risk represents the sum of the NSSI and suicide attempt scores from the BSL-23 Supplement. Δ vs Ctrl indicates the difference between a treatment and the control in change score at each timepoint relative to baseline scores. Cohen’s d is the effect size for Δ vs Ctrl. One asterisk (*) indicates significance at the p < 0.05 level; two asterisks indicate significance at p < 0.01. Time B = Day 15: post psychoeducation & prior to feedback, Time C = Day 30: post feedback, Time D = Day 60. | | | | | |

Reviewer #4: Thank you for the opportunity to review the manuscript entitled "Online psychoeducation and assessment for borderline personality disorder as a first step of care: A pilot study assessing safety, feasibility, and mechanisms of change". This is a timely study that warrants more attention from the field of mental healthcare. Below are my comments regarding the current manuscript:

1. Please cite the reference for the characteristic traits of BPD (line 67-69).

**Thank you for catching that- the DSM-5 was cited on line 69 in the Introduction of the manuscript.**

2. Please elaborate on the personal & societal costs mentioned (line 71-73).

**We have done so:**

**These costs result not only from direct care, but also from damaging reckless impulsivity, lost productivity, and informal or community care compounding to 62,260 euros (i.e., approximately $72,000) per patient reported annually [4].**

3. Please provide evidence for the claim regarding availability of recommended therapies for BPD (75-85).

**We have updated the Introduction:**

**Yet such treatment is rarely the first step of care [7]. Even in countries best resourced with specialized psychotherapies for BPD, only a minority of patients will receive psychotherapy immediately after diagnosis due to the restricted supply of trained professionals available to provide them [8]. Worldwide, the availability of recommended therapies for BPD is grossly insufficient, leaving people with a disorder defined by frantic efforts to avoid being abandoned at risk for self-injury and destructive interpersonal dynamics without alternatives for care. Our own study using publicly available data for healthcare workforce combined with records of specialist therapy certification bodies in 22 countries indicated that no country possessed an adequate supply of certified specialists to meet demands [8]. Even in nations with enriched specialist personality disorder services such as the Netherlands, psychotherapy is rarely the first step of care, despite being the primary recommended treatment for the diagnosis of BPD [7].**

4. Please provide the internal consistency of the self-developed Knowledge of BPD scale.
**Thank you- see responses to Reviewer 1 item 6 who had the same request.**

5. I wonder if there are any qualitative data that can be triangulated to further enhance the quantitative findings.

**Apologies there is no qualitative data we collected because this was fully online with surveys and performance tasks, but limited open ended responses, except on EMA where there are “other” responses.**
