# Supplementary material for: Online psychoeducation and assessment for borderline personality disorder as a first step of care: A pilot study assessing safety, feasibility, and mechanisms of change
Source: PLoS One. 2026 Aug 19;21(8):e0353836. doi: 10.1371/journal.pone.0353836 (PMC13489446; doi:10.1371/journal.pone.0353836)
Supplement: Peer review history

# Peer review: Decision Letter 0

**DOI:** 10.1371/journal.pone.0353836.r001

**Authors:** Hong Wang Fung

15 Apr 2026

Dear Dr. Choi-Kain,

Thank you for submitting your manuscript to PLOS ONE. After careful consideration, we feel that it has merit but does not fully meet PLOS ONE’s publication criteria as it currently stands. Therefore, we invite you to submit a revised version of the manuscript that addresses the points raised during the review process.

==============================

Thank you for submitting your very interesting manuscript. Please address the reviewers' comments.

==============================

Please submit your revised manuscript by May 30 2026 11:59PM. If you will need more time than this to complete your revisions, please reply to this message or contact the journal office at plosone@plos.org. When you're ready to submit your revision, log on to https://www.editorialmanager.com/pone/ and select the 'Submissions Needing Revision' folder to locate your manuscript file.

- A letter that responds to each point raised by the academic editor and reviewer(s). You should upload this letter as a separate file labeled 'Response to Reviewers'.
- A marked-up copy of your manuscript that highlights changes made to the original version. You should upload this as a separate file labeled 'Revised Manuscript with Track Changes'.
- An unmarked version of your revised paper without tracked changes. You should upload this as a separate file labeled 'Manuscript'.

If you would like to make changes to your financial disclosure, please include your updated statement in your cover letter. Guidelines for resubmitting your figure files are available below the reviewer comments at the end of this letter.

If applicable, we recommend that you deposit your laboratory protocols in protocols.io to enhance the reproducibility of your results. Protocols.io assigns your protocol its own identifier (DOI) so that it can be cited independently in the future. For instructions see: https://journals.plos.org/plosone/s/submission-guidelines#loc-laboratory-protocols. Additionally, PLOS ONE offers an option for publishing peer-reviewed Lab Protocol articles, which describe protocols hosted on protocols.io. Read more information on sharing protocols at https://plos.org/protocols?utm_medium=editorial-email&utm_source=authorletters&utm_campaign=protocols.

As the corresponding author, your ORCID iD is verified in the submission system and will appear in the published article. PLOS supports the use of ORCID, and we encourage all coauthors to register for an ORCID iD and use it as well. Please encourage your coauthors to verify their ORCID iD within the submission system before final acceptance, as unverified ORCID iDs will not appear in the published article. *Only*  the individual author can complete the verification step; PLOS staff *cannot*  verify ORCID iDs on behalf of authors.

We look forward to receiving your revised manuscript.

Kind regards,

Hong Wang Fung

Academic Editor

PLOS One

**Journal Requirements:**

When submitting your revision, we need you to address these additional requirements.

1. Please ensure that your manuscript meets PLOS ONE's style requirements, including those for file naming. The PLOS ONE style templates can be found at

https://journals.plos.org/plosone/s/file?id=wjVg/PLOSOne_formatting_sample_main_body.pdf and

https://journals.plos.org/plosone/s/file?id=ba62/PLOSOne_formatting_sample_title_authors_affiliations.pdf

2. Please include your tables as part of your main manuscript and remove the individual files. Please note that supplementary tables (should remain/ be uplo`aded) as separate "supporting information" files.

3. We note that the grant information you provided in the ‘Funding Information’ and ‘Financial Disclosure’ sections do not match.

When you resubmit, please ensure that you provide the correct grant numbers for the awards you received for your study in the ‘Funding Information’ section.

4. In the online submission form you indicate that your data is not available for proprietary reasons and have provided a contact point for accessing this data. Please note that your current contact point is a co-author on this manuscript. According to our Data Policy, the contact point must not be an author on the manuscript and must be an institutional contact, ideally not an individual. Please revise your data statement to a non-author institutional point of contact, such as a data access or ethics committee, and send this to us via return email. Please also include contact information for the third party organization, and please include the full citation of where the data can be found.

5. Please include your full ethics statement in the ‘Methods’ section of your manuscript file. In your statement, please include the full name of the IRB or ethics committee who approved or waived your study, as well as whether or not you obtained informed written or verbal consent. If consent was waived for your study, please include this information in your statement as well.

6. Please upload a new copy of Figures 2-3, and Supporting Information Table 1 as the detail is not clear. Please follow the link for more information:  https://journals.plos.org/plosone/s/figures

7. We note that the original protocol that you have uploaded as a Supporting Information file contains an institutional logo. As this logo is likely copyrighted, we ask that you please remove it from this file and upload an updated version upon resubmission.

8. If the reviewer comments include a recommendation to cite specific previously published works, please review and evaluate these publications to determine whether they are relevant and should be cited. There is no requirement to cite these works unless the editor has indicated otherwise.

**Additional Editor Comments:**

Thank you for submitting your very interesting manuscript. Please address the reviewers' comments.

[Note: HTML markup is below. Please do not edit.]

Reviewers' comments:

Reviewer's Responses to Questions

**Comments to the Author**

1. Is the manuscript technically sound, and do the data support the conclusions?

Reviewer #1: Partly

Reviewer #2: Yes

Reviewer #3: Partly

Reviewer #4: Yes

**********

2. Has the statistical analysis been performed appropriately and rigorously?

Reviewer #1: No

Reviewer #2: Yes

Reviewer #3: No

Reviewer #4: Yes

**********

3. Have the authors made all data underlying the findings in their manuscript fully available?

The PLOS Data policy

Reviewer #1: Yes

Reviewer #2: Yes

Reviewer #3: Yes

Reviewer #4: Yes

**********

4. Is the manuscript presented in an intelligible fashion and written in standard English?

Reviewer #1: Yes

Reviewer #2: Yes

Reviewer #3: Yes

Reviewer #4: Yes

**********

Reviewer #1: This study addresses an important and challenging population and demonstrates several notable strengths. The inclusion of lived experience input is particularly valuable. Research with individuals with BPD is inherently difficult due to ethical considerations and risk management, which complicates recruitment, retention, and safety monitoring. Despite these challenges, the authors successfully implemented a fully online intervention and assessment protocol, demonstrating feasibility and acceptability in a population that is often hard to engage.

Nonetheless, several methodological and reporting issues limit interpretation and generalizability. The authors appear aware of these limitations, and the preliminary data are promising. Please see the attached comments for specific revisions and clarifications regarding diagnosis verification, intervention adherence, measurement, safety monitoring, statistical approach, attrition, and potential confounds. I look forward to seeing this work progress.

Reviewer #2: This RCT takes an interesting and possibly very useful concept of using virtual tools to ameliorate the impact of BPD prior to receiving long term treatment.

Overall it is well written and comprehensive in describing methods, and results.

Most of the concerns I had have been addressed in the limitation section.

One point to clarify is when you included those that had taken some form or duration of DBT and MBT did you consider the possible confounding effect on the response to the psycho education (with or without feedback)

Also consider summarizing the introduction, it’s a bit long.

Reviewer #3: This is a feasibility and safety of a randomized controlled trial protocol of online psychoeducation and personalized

feedback as an immediate first step of care for BPD.

Some comments for the authors:

1) While at this stage it might be late - consider title incoporates that this is randomised feasibility study - for searchability.

2) They are some differences between pilot and feasibility studies and these are used interchangeably in the manuscript - can this is be clarified. For example, feasibility studies usually assess parameters such as recruitment,retention,acceptability of intervention (.i.e as you have noted in lines 317 - 326). Pilot studies are essentially a study, e.g mimicing the larger study - however in this manuscript there seems to a combination of both with some statistical formal testing which seems inappropriate.

3) There seems to be no justification of the number of people (sample size) for the study - even though they are planned significance tests

4) Can there more information around the randomisation process (lines 243 - 248 talks about having fixed parameters to ensure balance - but this would be determined in the computergenerated scehedule, i.e having three groups with stratification as well - can more information be added to explain the rationale for this - or more clarity please. how was allocation concealment achieved?

5) Assuming this is RCT feasibility study - there should be no between group differences at baseline since any differences are due to chance.

6) Was there a statistical analysis plan, prior to final analyses,

7) Any independent committee reviewing/monitoring safety of these participants.

8) How was missing data handled?

9) Table 1 - all baseline symptoms, can you also report other metrics, i.e median range - since sample sizes are small, removed p-values

10) Table 3 - Primary outcomes - firstly maybe have a foot note for meaning og Time B, C, D and really the primary outcomes of the feasibility studies should be the ones mentioned, i.e recruitment etc - can this be clarified.

Reviewer #4: Thank you for the opportunity to review the manuscript entitled "Online psychoeducation and assessment for borderline personality disorder as a first step of care: A pilot study assessing safety, feasibility, and mechanisms of change". This is a timely study that warrants more attention from the field of mental healthcare. Below are my comments regarding the current manuscript:

1. Please cite the reference for the characteristic traits of BPD (line 67-69).

2. Please elaborate on the personal & societal costs mentioned (line 69-70).

3. Please provide evidence for the claim regarding availability of recommended therapies for BPD (75-78).

4. Please provide the internal consistency of the self-developed Knowledge of BPD scale.

5. I wonder if there are any qualitative data that can be triangulated to further enhance the quantitative findings.

**********

what does this mean?). If published, this will include your full peer review and any attached files.

If you choose “no”, your identity will remain anonymous but your review may still be made public.

**Do you want your identity to be public for this peer review?**  For information about this choice, including consent withdrawal, please see our Privacy Policy

Reviewer #1: No

Reviewer #2: No

Reviewer #3: No

Reviewer #4: No

**********

[NOTE: If reviewer comments were submitted as an attachment file, they will be attached to this email and accessible via the submission site. Please log into your account, locate the manuscript record, and check for the action link "View Attachments". If this link does not appear, there are no attachment files.]

To ensure your figures meet our technical requirements, please review our figure guidelines: https://journals.plos.org/plosone/s/figures

You may also use PLOS’s free figure tool, NAAS, to help you prepare publication quality figures: https://journals.plos.org/plosone/s/figures#loc-tools-for-figure-preparation.

NAAS will assess whether your figures meet our technical requirements by comparing each figure against our figure specifications.

Attachment Submitted filename: additional_comments.pdf

# Author comment: Author response to Decision Letter 1

**DOI:** 10.1371/journal.pone.0353836.r002

4 Jun 2026

Dear Editors of PLOS One and Reviewers:

We thank you for your thorough and thoughtful review of our manuscript. We feel the feedback has improved and tightened our report. Please see the responses to each item below.

Reviewer #1: This study addresses an important and challenging population and demonstrates several notable strengths. The inclusion of lived experience input is particularly valuable. Research with individuals with BPD is inherently difficult due to ethical considerations and risk management, which complicates recruitment, retention, and safety monitoring. Despite these challenges, the authors successfully implemented a fully online intervention and assessment protocol, demonstrating feasibility and acceptability in a population that is often hard to engage.

Thank you for appreciating the challenges of a fully online randomized controlled trial. The fact that it was fully online with no face-to-face interaction or live virtual interactions after screening may explain some of the limitations in methodology brought up in the reviewer’s comments.

Nonetheless, several methodological and reporting issues limit interpretation and generalizability. The authors appear aware of these limitations, and the preliminary data are promising. Please see the attached comments for specific revisions and clarifications regarding diagnosis verification, intervention adherence, measurement, safety monitoring, statistical approach, attrition, and potential confounds. I look forward to seeing this work progress.

Additional comments

1. BPD diagnosis a. How many participants were formally diagnosed vs self-diagnosed? b. Across recruitment sources, how was diagnosis confirmed (structured interview, clinician report, solely self-report)?

All participants had to answer the following question during the Screening Questionnaire, “Were you diagnosed with borderline personality disorder (BPD) by a (mental) health care provider (e.g., primary care physician, psychologist, psychiatrist, social worker, or licensed mental health counselor) within the past six months?” All participants except for two self-reported being formally diagnosed by a health provider. Of these 80 participants, 48.8% were diagnosed by a clinician during inpatient psychiatric treatment, 46.2% were diagnosed by an outpatient mental health professional, 2.5% by a doctor in the emergency room, and 2.5% by a primary care physician. We had no way of verifying this information as we only had one live screening interview and no subsequent live interaction.

A Welch two-sample t-test showed no evidence of a significant difference in baseline BSL-23 scores between self-diagnosed and formally participants (t = 0.02, df = 2.24, p = .99). Mean scores were nearly identical between groups (Self: 2.30; Formal: 2.31). That said, the accuracy for this t-test is limited given the extremely low sample size in the self-diagnosed subgroup. Of the two participants who reported a self-diagnosis of BPD, one had a baseline BSL score of 2.43 and the other a score of 2.17, with both values falling within the high severity category.

Diagnosis was based solely on self-report, which we discuss as a limitation in the Discussion section of the manuscript on lines 784-790. However, baseline BSL-23 scores indicated that all participants scored above the typical range observed in healthy control samples, suggesting clinically relevant symptom levels. In this sample, the minimum baseline BSL-23 score was 0.74 compared to the 0.64 diagnostic threshold. We report the mean baseline BSL-23 score in the Limitations subsection of the Discussion section, and the minimum and maximum baseline scores are depicted in Table 1.

While this is not as reliable as a semi-structured interview method of diagnosis or clinical diagnosis, it is what we could complete with a total budget from this pilot study grant of $75K. Future definitive studies will employ more rigorous verification of diagnosis.

2. The CONSORT diagram excludes DBT/MBT, but the text states inclusion criteria were later expanded to allow some prior exposure. Please reconcile this discrepancy.

This discrepancy was noted in the legend of Fig 1 (CONSORT diagram). We also revised the legend for Fig 1 to clarify that the recruitment criteria were later expanded to extend eligibility beyond Massachusetts, given the online nature of the study.

a. What were the definitions of “partial” versus “full” exposure? b. How was adherence to previous therapy determined?

Research assistants performed all eligibility screening which was reviewed with the PI and first author to determine if exposure was significant or not. There were no means available to qualify or assess prior exposures since this trial did not conduct or supervise those treatments which were not video-taped or rated.

16 participants reported experience with evidence-based treatment (DBT, MBT, or TFP) in the Screening Questionnaire, but a total of 36 participants reported prior exposure to DBT (N=32), MBT (N=3), or TFP (N=1) during the formal Treatment History Inventory during the baseline assessment.

Provided below are what participants reported:

• DBT - 2 partial hospitalization programs outpatient 2 weeks (Salem hospital)

• DBT in outpatient programs

• DBT skills with outpatient therapist, never discussed BPD

• DBT in a partial program as a teenager

• DBT group (1 year) through an MGB hospital, not standard full package DBT

• did “some” DBT at McLean which does not offer adherent DBT in the general adult outpatient clinic, only groups

• 4 participants did not provide information about what constituted their experience with prior EBTs in the Screening Questionnaire.

Partial exposure to evidence-based treatment was defined as limited engagement with a therapy without completing the full course as studied in prior RCTs. For example, one participant attended a two-week outpatient program that incorporated DBT-informed interventions, while another received DBT skills training from an outpatient therapist.

Information regarding how we defined full exposure to evidence-based treatment is reported in the Participants subsection of the Materials and Methods section of the manuscript (lines 212-214).

3. Considering BPD severity is a strength of this study. a. How were the “high” vs “low” categories defined? b. What was the pre-determined threshold?

Severity was based on baseline BSL scores, with a cut-off of 2.67. This information is reported in the Design subsection of the Materials and Methods section of the manuscript and was determined by the severity ranges indicated in prior studies establishing those standards (lines 238-239). Severity cutoffs for average score variable are: none/low (0-.28), mild (.28-1.07), moderate (1.07-1.87), high (1.87-2.67), very high (2.67-3.47), extremely high (3.47-4.0). Cutoff for diagnostic BPD is 1.50 compared to clinical controls and .64 compared to healthy controls.

These severity cutoffs were introduced and validated in the following paper: Kleindienst N, Jungkunz M, Bohus M. A proposed severity classification of borderline symptoms using the borderline symptom list (BSL-23). Borderline personality disorder and emotion dysregulation. 2020 Jun 1;7(1):11.

4. How was it verified that participants watched the videos (clicks, time spent, self-report)? a. Could videos be watched multiple times? b. If a participant missed a day, could/did they watch two videos the next day?

With our limited budget and low-tech method, we could not verified what participants watched or not, which is addressed as a limitation in the Limitations section of the Discussion on lines 790-792. However, the patient knowledge test of BPD information used assessed some items directly covered in the videos that suggest they had been watched. See answer to psychometric characterization of our knowledge test which was specifically developed for this study.

Yes, it is possible participants watched the videos multiple times. Yes, it was possible for participants to watch videos later. There was no timeframe in which video access was turned off.

5. Feedback is a convenient term for the additional component the feedback group received, but can it truly be considered purely informational – or did it likely function as an intervention alongside the videos? (particularly in relation to improvements in LPFS scores for the feedback group)

Please see supporting information S1 File Example of Feedback for your review so that you and readers can see the simplicity as well as impact of this automated feedback. These feedback documents were derived directly from participant self-report and cognitive testing data, and explanation of the data, and a summary of improvements, things that remained steady, and things that worsened. We hypothesized that measurement and feedback itself had therapeutic effects, and this part of the design is described in the prior protocol paper we published in this journal in 2023.

6. The BPD knowledge assessment appears closely tied to intervention content, raising potential “teaching to the test” concerns, especially since the 13-item multiple-choice measure was developed by the study team based on the BPD psychoeducational videos. a. Is there any reliability or psychometric data, or is this a non-validated measure? b. Were ceiling effects considered or observed?

We have added psychometric elaboration of this new measure (Patient Knowledge Test- Borderline Personality Disorder: PKT-BPD) we are including in Supporting Information S2 File BPD Knowledge Test. We have also added a reliability analysis using Cronbach’s alpha indicated suboptimal internal consistency across all timepoints (Time Point A: α = 0.50; Time Point B: α = 0.61; Time Point D: α = 0.62). Although reliability increased slightly over time, values remained below conventional thresholds for acceptable internal consistency. Cronbach’s alpha assumes one-dimensionality and item homogeneity. Violations of these assumptions, such as those common in knowledge-based assessments with heterogeneous content, can result in lower alpha values without necessarily indicating poor measurement quality (Edelsbrunner, Simonsmeier, & Schneider, 2025). See lines 327-338.

We also analyzed floor and ceiling effects as suggested. Our analysis indicated no floor effects at any timepoint, suggesting that the measure was not too difficult for participants and that minimum scores were not reached. In contrast, ceiling effects were observed and increased over time, indicating that a growing proportion of participants achieved the maximum possible score at follow-up assessments.

When examined by condition, the control group showed a modest ceiling effect at baseline (10.7%) that was not observed at later timepoints, suggesting stable and relatively limited BPD knowledge. The psychoeducation group demonstrated minimal ceiling effects at baseline (4%), which increased at Time Point B (27.8%) before decreasing at Time Point D (14.3%), indicating an initial increase in knowledge followed by a reduction in scores over time. The psychoeducation + feedback group showed the strongest ceiling effects, increasing from 3.45% at baseline to 28.6% at Time Point B and 35% at Time Point D, suggesting substantial knowledge gains throughout the study. We have added this in lines 504-516. The PKT-BPD measure can be found in Supporting Information S2 and the Table for the ceiling and floor effects can be found at S2 Table.

c. Was participant self-directed education measured? (i.e., research participants did unrelated to the study)

Self-directed psychoeducation was not tracked as we had no way of monitoring this aside from self-report.

7. Defining safety solely as ED visits, hospitalization, or residential care may be limited for a BPD population. a. Were individual “high-risk” behaviors tracked separately (e.g., could self-harm be analyzed as its own variable, or was it combined with other high-risk behaviors)?

The BSL-23 Supplement for Assessing Behaviors inquiries about the frequency of various high-risk behaviors in the past week, including suicide attempts and non-suicidal self-injury (NSSI). A composite risk variable was created using the NSSI and suicide attempt items from the BSL–23 Supplement, with higher scores reflecting greater frequency of these behaviors. Descriptive statistics for the composite risk variable are presented in Table 4, which is reported in the BPD Symptoms subsection of the Measures section (lines 347-350).

A linear mixed effects model with random intercepts for participant revealed that neither time (F = 0.42; p = .64), condition (F = 0.32, p = .88), nor the time x condition interaction (F = 1.14, p = .34) significantly predicted risk. In addition, a Poisson mixed effects model demonstrated that baseline risk did not significantly predict dropout (β = -0.011, p = .55), which is reported in the Change in Overall BPD Symptoms subsection of the Results (lines 542-547).

b. Is including step-up to residential care appropriate, given that not all programs allow phone access, which could affect attrition?

In the present study, neither stepping up to residential care nor being hospitalized affected participation. The one participant who reported initiating residential treatment in the past 15 days and dropped out, continued in the study after entering residential treatment at time point B with completed data for that time point, but withdrew prior to time point C. Of the eight participants who reported hospitalization in the past 15 days, only one dropped out later on, specifically this participant was hospitalized prior to time point B and dropped out before time point D, having completed data through time point C.

Originally, one participant reported the same hospitalization event at both time points B and C. In addition, another participant reported entering residential care at time point B, but the reported date preceded completion of the screening questionnaire. Both participants’ responses were therefore recoded, resulting in changes to Table 2.

Fisher’s exact test indicated no statistically significant association between escalating care, either hospitalization or entering residential treatment (odds ratio = 1.3, 95% CI [0.02, 14.9], p = 1.00) and drop out status, though the estimate was imprecise, as reflected by the wide confidence interval due to the small number of events.

8. Treating ordinal responses as integers and averaging across observations for the regression linking BSL change to time spent alone may be a crude approximation. Please justify this approach or consider alternatives that respect the ordinal nature, such as ordinal mixed models or cumulative link models.

We would like to first clarify that the ordinal variable (time spent alone, EMA-assessed on a 1 to 4 scale) enters our regression as a predictor, with BSL change at Time C as the continuous outcome. The reviewer's suggested alternatives, cumulative link models and ordinal mixed models, are designed for ordinal outcomes and thus are not directly applicable here, which is why those methods were not adopted.

Second, the EMA time-spent-alone item is best understood as a coarsened continuous variable rather than a qualitatively ordered categorical one. Response options correspond to binned hours: 1 = "less than 1 hour," 2 = "1–3 hours," 3 = "4–6 hours," 4 = "7 or more hours." The category midpoints (~0.5, 2, 5, ~10 hours) are monotonically ordered and approximately linear with respect to the integer codes, so the 1-4 coding serves as a reasonable linear approximation of the actual hours spent alone. Averaging the integer codes across 30 observations therefore provides a close approximation to each participant's actual mean hours of time spent alone, and preserves the ordering of participants on their typical level of time spent alone.

9. Attrition is substantial (27 participants, 32.9%). Dropout may not be a robust proxy for a measure of acceptability, since participants may have discontinued for a variety of reasons.

Thank you for this point, we agree. This attrition rate is relatively high but still falls within the bounds of what is to be expected from patients with BPD

Attachment Submitted filename: Response to Reviewers FINAL.docx

# Peer review: Decision Letter 1

**DOI:** 10.1371/journal.pone.0353836.r003

**Authors:** Hong Wang Fung

30 Jun 2026

Online psychoeducation and assessment for borderline personality disorder as a first step of care: A pilot study assessing safety, feasibility, and mechanisms of change

PONE-D-26-11438R1

Dear Dr. Choi-Kain,

We’re pleased to inform you that your manuscript has been judged scientifically suitable for publication and will be formally accepted for publication once it meets all outstanding technical requirements.

Within one week, you’ll receive an e-mail detailing the required amendments. When these have been addressed, you’ll receive a formal acceptance letter and your manuscript will be scheduled for publication.

An invoice will be generated when your article is formally accepted. Please note, if your institution has a publishing partnership with PLOS and your article meets the relevant criteria, all or part of your publication costs will be covered. Please make sure your user information is up-to-date by logging into Editorial Manager at Editorial Manager® and clicking the ‘Update My Information' link at the top of the page. For questions related to billing, please contact billing support.

If your institution or institutions have a press office, please notify them about your upcoming paper to help maximize its impact. If they’ll be preparing press materials, please inform our press team as soon as possible -- no later than 48 hours after receiving the formal acceptance. Your manuscript will remain under strict press embargo until 2 pm Eastern Time on the date of publication. For more information, please contact onepress@plos.org.

Kind regards,

Hong Wang Fung

Academic Editor

PLOS One

Additional Editor Comments (optional):

Our reviewers have now reviewed your revised submission and recommended publication in its current form. Thank you for your contributions.

Reviewers' comments:

Reviewer's Responses to Questions

**Comments to the Author**

Reviewer #3: All comments have been addressed

**********

2. Is the manuscript technically sound, and do the data support the conclusions?

Reviewer #3: Yes

**********

3. Has the statistical analysis been performed appropriately and rigorously?

Reviewer #3: Yes

**********

4. Have the authors made all data underlying the findings in their manuscript fully available?

The PLOS Data policy

Reviewer #3: Yes

**********

5. Is the manuscript presented in an intelligible fashion and written in standard English?

Reviewer #3: Yes

**********

Reviewer #3: All commentd addressed.

**********

what does this mean?). If published, this will include your full peer review and any attached files.

If you choose “no”, your identity will remain anonymous but your review may still be made public.

**Do you want your identity to be public for this peer review?**  For information about this choice, including consent withdrawal, please see our Privacy Policy

Reviewer #3: No

**********

# Editor report: Acceptance letter

**DOI:** 10.1371/journal.pone.0353836.r004

**Authors:** Hong Wang Fung

PONE-D-26-11438R1

PLOS One

Dear Dr. Choi-Kain,

I'm pleased to inform you that your manuscript has been deemed suitable for publication in PLOS One. Congratulations! Your manuscript is now being handed over to our production team.

At this stage, our production department will prepare your paper for publication. This includes ensuring the following:

* All references, tables, and figures are properly cited

* All relevant supporting information is included in the manuscript submission,

* There are no issues that prevent the paper from being properly typeset

You will receive further instructions from the production team, including instructions on how to review your proof when it is ready. Please keep in mind that we are working through a large volume of accepted articles, so please give us a few days to review your paper and let you know the next and final steps.

Lastly, if your institution or institutions have a press office, please let them know about your upcoming paper now to help maximize its impact. If they'll be preparing press materials, please inform our press team within the next 48 hours. Your manuscript will remain under strict press embargo until 2 pm Eastern Time on the date of publication. For more information, please contact onepress@plos.org.

You will receive an invoice from PLOS for your publication fee after your manuscript has reached the completed accept phase. If you receive an email requesting payment before acceptance or for any other service, this may be a phishing scheme. Learn how to identify phishing emails and protect your accounts at https://explore.plos.org/phishing.

If we can help with anything else, please email us at customercare@plos.org.

Thank you for submitting your work to PLOS ONE and supporting open access.

Kind regards,

PLOS ONE Editorial Office Staff

on behalf of

Dr. Hong Wang Fung

Academic Editor

PLOS One
